# Supplementary material for: In vitro antiproliferative and apoptotic effects of thiosemicarbazones based on (-)-camphene and R-(+)-limonene in human melanoma cells
Source: PLoS One. 2023 Nov 30;18(11):e0295012. doi: 10.1371/journal.pone.0295012 (PMC10688736; doi:10.1371/journal.pone.0295012)
Supplement: S1 File — Structural characterization by 1H and 13C NMR of the new (-)-camphene-based compounds. (PDF) [file pone.0295012.s001.pdf]

S1 Structural characterization of the new thiosemicarbazone derivatives

S1.1 Chemical characterization

The ^{1}H and ^{13}C NMR spectra were acquired using a VARIAN Mercury Plus spectrometer operating at 300 MHz for ^{1}H and 75.5 MHz for ^{13}C, with tetramethylsilane (TMS) or the respective solvent serving as the internal reference. Chemical shifts are reported in parts per million (ppm), and $CHCl^3$ was employed as the solvent**.** The absorption spectra in the IR region were recorded using a BOMEM MB-100 spectrophotometer, with a KBr pellet, in the range of 400 to 4000 cm^{-1}. Low-resolution mass spectra were obtained using a SHIMADZU-QP 2000A mass spectrometer, with a 70 eV electron beam, equipped with a solid sample probe. Melting points were determined using the Microquímica MQAPF-301 apparatus. Optical rotations were measured in a $CHCl_3$ solution with a Perkin Elmer model 343 polarimeter at 25ºC.

The data presented correspond to the new compounds. Information for the other studied compounds has been previously published and is available in the references provided in the Materials and Methods section.

***p-M*ethyl-benzaldehyde (-)-camphene- based thiosemicarbazone (compound 5 in Fig 1)**

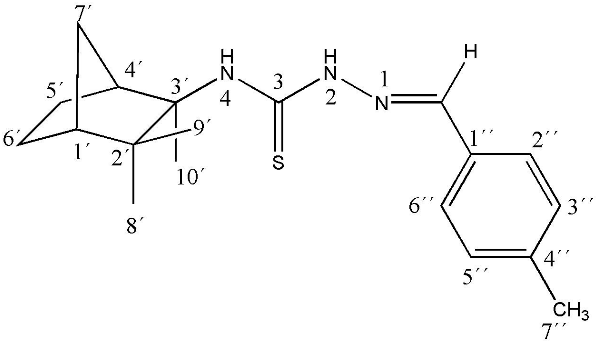

White crystals; yield 94%; mp. 219-221ºC; $[\alpha]_D$ +33; IR (KBr/cm^{-1}): (NH) 3339 and 3150, (C=S) 1340; EI-MS m/z 329 ($M^{+\bullet}$); ^{1}H NMR (300 MHz, $CDCl_3$): δ_H 1.07 (3H, s, H-10'),1.31 (3H, s, H-9'),1.74 (3H, s, H-8'),1.52 (2H, m, H-5'), 1.60 (2H, m, H-6′), 1.21-1.85 (2H, m, H-7'), 1.80 (1H, d,J=2.4, H-1'), 2.27 (3H, s, H-7"), 3.26 (1H, d, J=3.0, H-4'), 7.19 (2H, d, J=8.7, H-3"/ H-5"), 7.92 (1H, s, C=N, H-1"), 7.48 (2H, d, J=8.7, H-2" /H-6"), 8.03 (1H, sl, H-4), 8.91 (1H, sl, NH, H-2); ^{13}C NMR (75.5 MHz, $CDCl_3$): δ 18.11 (C-8′),23.59 (C-10'), 26.45 (C9'), 23.48 (C-6'), 23.51 (C-5'), 35.02 (C-7'), 45.91 (C-2'), 48.06 (C-4'), 50.44 (C-1\`), 66.62 (C-3'), 127.20 (C-3"/ C-5"), 129.77 (C-2"/C-6"), 21.69 (C-7"), 131.05 (C-1"), 142.2 (C=N), 140.76 (C-4"), 175.33(C=S, C-3).

m-Chloro-benzaldehyde (-)-camphene-based thiosemicarbazone (compound 8 in Fig 1)

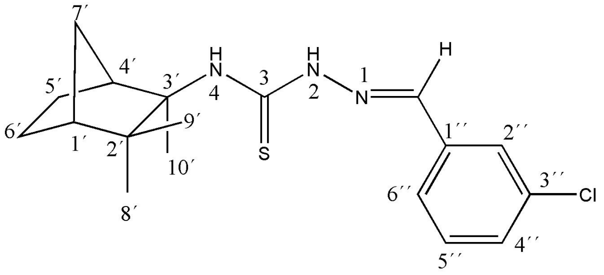

White crystals; yield 86%; mp. 145-147ºC; $[\alpha]_D$ +18; IR (KBr/cm^{-1}): (NH) 3288 and 3141, (C=S) 1391; EI-MS m/z 349 ($M^{+\bullet}$); ^{1}H NMR (300 MHz, $CDCl_3$): δ_H 1.07 (3H, s, H-10’), 1.32 (3H, s, H-9’), 1.74 (3H, s, H-8’), 1.45 (2H, m, H-5’), 1.59 (2H, m, H-6′), 1.18-1.86 (2H, m, H-7’), 1.80 (1H, d, J=3.3, H-1’), 3.30 (1H, d, J=3.0, H-4’), 7.33 (2H, d, H3”/ H-4”), 7.45 (2H, d, H2”), 7.58 (2H, d, H6”), 7.92 (1H, s, C=N, H-1”), 8.07 (1H, sl, H-4), 10.39 (1H, sl, NH, H-2);^{13}C NMR (75.5 MHz, $CDCl_3$): δ 18.02 (C-8′), 23.57 (C-10’), 26.33 (C9’), 23.45 (C-6’), 23.48 (C-5’), 34.87 (C-7’), 45.88 (C-2’), 50.36 (C-1’), 47.84 (C-4’), 66.71 (C-3’), 130.14(C3”); 135.04(C5”), 130.14(C-4”) (C-2”), 135.77(C-1”), 139.67 (C=N), 175.20 (C=S, C-3).

o-Nitro-benzaldehyde (-)-camphene-based thiosemicarbazone (compound 10 in Fig 1)

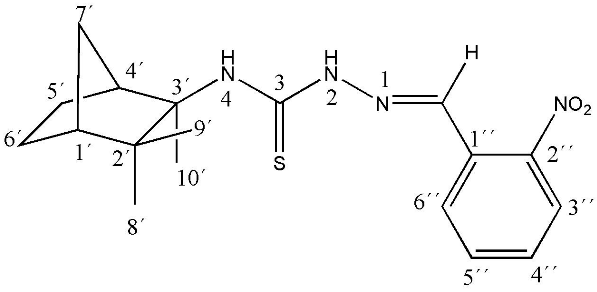

Yellow crystals; yield 84%; mp. 135-138ºC; $[\alpha]_D$ +40; IR (KBr/cm^{-1}): (NH) 3336 and 3139, (C=S) 1341; EI-MS m/z 360 ($M^{+\bullet}$); ^{1}H NMR (300 MHz, $CDCl_3$): δ_H 1.07 (3H, s, H-10’),1.31 (3H, s, H-9’),1.73 (3H, s, H-8’), 1.48 (2H, m, H-5’), 1.60 (2H, m, H-6′), 1.18-1.62 (2H, m, H-7’), 1.80 (1H, d, J=2.4, H-1’), 3.23 (1H, d, J=3.6, H-4’), 7.61 (2H, d, H-4”), 7.65 (2H, d, H-3”), 7.96 (2H, d, J=7.2, H-5”), 7.85 (1H, s, C=N, H-1”), 7.48 (2H, d, J=8.7), 8.05 (1H, sl, H-4), 10.01 (1H, sl, NH, H-2);^{13}C NMR (75.5 MHz, $CDCl_3$): δ 18.00 (C-8′), 23.32 (C-10’), 26.30 (C9’), 23.32 (C-6’), 23.32 (C-5’), 34.87 (C-7’), 45.80 (C-2’), 50.30 (C-1’), 48.00 (C-4’), 66.40 (C-3’), 133,17 (C-3”), 128.86 (C-2”), 124,85 (C-5”), 148.28 (C-6”), 128.36 (C-1”), 140.06 (C=N), 130.35 (C-4”), 175.79(C=S, C-3).

***m*-Nitro-benzaldehyde (-)-camphene-based thiosemicarbazone (compound 11 in Fig 1)**

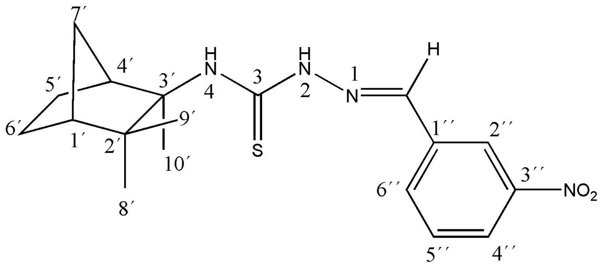

Yellow crystals; yield 91%; mp. 126-128ºC; $[\alpha]_D$ +51; IR (KBr/cm^{-1}): (NH) 3345 and 3142, (C=S) 1348; EI-MS m/z 360 ($M^{+\bullet}$); ^{1}H NMR (300 MHz, $CDCl_3$): δ_H 1.07 (3H, s, H-10'),1.31 (3H, s, H-9'),1.73 (3H, s, H-8'), 1.48 (2H, m, H-5'), 1.60 (2H, m, H-6′), 1.18-1.62 (2H, m, H-7'), 1.80 (1H, d, J=2.4, H-1'), 3.23 (1H, d, J=3.6, H-4'), 7.74 (2H, d, J=8.7, H3"/ H-5"), 8.26 (2H, d, J=8.7, H2"/H-6"), 8.16 (1H, s, C=N, H-1"), 8.49 (1H, sl, H-4), 10.80 (1H, sl, NH, H-2); ^{13}C NMR (75.5 MHz, $CDCl_3$): δ 18.00 (C-8′), 23.32 (C-10'), 26.30 (C9'), 23.32 (C-6'), 23.32 (C-5'), 34.87 (C-7'), 45.80 (C-2'), 50.30 (C-1'), 48.00 (C-4'), 66.40 (C-3'), 127.62 (C-3"/C5"), 124.39 (C-2"/C6"), 125.30(C-1"), 136.24 (C=N), 174.76(C=S, C-3).

***m*-Methoxy-*p*-hydroxybenzaldehyde (-)-camphene-based thiosemicarbazone (compound 15 in Fig 1)**

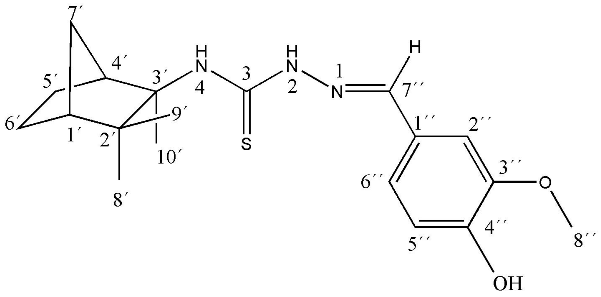

Yield 60%; EI-MS m/z 361.50 ($M^{+\bullet}$); ^{1}H NMR (300 MHz, $CDCl_3$): δ_H 1.33 (3H, s, H-10'), 1.07 (3H, s, H-9'), 1.74 (3H, s, H-8'), 1.43-1.60 (2H, m, H-5'/H-6′), 1.18-1.85 (2H, m, H-7'), 1.80 (1H, m, H-1'), 3.91 (3H, s, H8"), 3.42 (1H, m, H-4'), 7.20 (1H, d, J=1.8, H-2"), 6.94 (1H, d, J=8.1, H-5"), 7.05 (1H, dd, J=8.1, H-6"), 7.68 (1H, d, H-7") 8.08 (1H, sl, H-4), 9.08 (1H, sl, NH, H-2); ^{13}C NMR (75.5 MHz, $CDCl_3$): δ 17.9 (C-8′), 26.2 (C-10'), 23.7 (C9'), 23.4 (C-6'), 23.6(C-5'), 35.0 (C-7'), 45.8 (C-2'), 47.4 (C-4'), 50.3 (C-1′), 66.6 (C-3'), 147.1 (C-3"), 148.1 (C-4"), 114.7 (C-5"), 122.7 (C-6"), 55.9 (C-8"), 126.2 (C-1"), 140.9 (C=N, C-7"), 148.1 (C-4"), 175.2 (C=S, C-3).

Acetophenone (-)-camphene-based thiosemicarbazone (compound 18 in Fig 1)

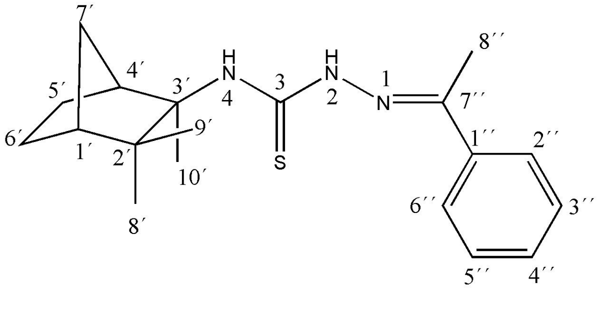

Yield 93%; EI-MS m/z 329.50 ($M^{+•}$); ^{1}H NMR (300 MHz, $CDCl_3$): δ_H 1.31 (3H, s, H-10'), 1.07 (3H, s, H-9'), 1.76 (3H, s, H-8'), 1.60 (2H, m, H-5'/H-6′), 1.19-1.87 (2H, m, H-7'), 1.79 (1H,m, H-1'),2.25 (3H, s, H8"), 3.24 (1H, d, H-4'), 2.25 (3H, s, H-8"), 7.68 (1H, m, H-3"/ H-5"), 7.39 (1H,m, H-2"),7.40 (1H, m, H-6"), 7.41 (1H, m, H-4"), 8.27 (1H, sl, H-4), 8.36 (1H, sl, NH, H-2); ^{13}C NMR (75.5 MHz, $CDCl_3$): δ 18.00 (C-8′),26.50 (C-10'), 23.60 (C9'), 23.40 (C-5′/C-6'), 35.00 (C-7'), 45.90 (C-2'), 48.10 (C-4'), 50.40 (C-1′), 66.70 (C-3'), 126.2 (C-3"/ C-5"), 128.87 (C-2"/C-6"), 13.4 (C-8"), 137.7 (C-1"), 145.10 (C=N, C-7"), 129.60 (C-4"), 176.1 (C=S, C-3).
IR (KBr/cm^{-1}): (NH) 3339 and 3150, (C=S) 1340

***p*-Nitroacetophenone (-)-camphene-based thiosemicarbazone (compound 19 in Fig 1)**

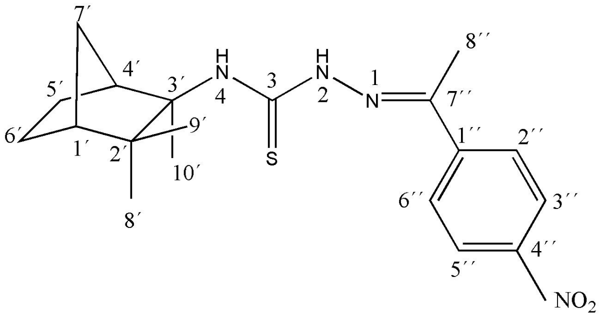

Yield 95%; EI-MS m/z 374.50 ($M^{+•}$); ^{1}H NMR (300 MHz, $CDCl_3$): δ_H 1.29 (3H, s, H-10'), 1.08 (3H, s, H-9'), 1.75 (3H, s, H-8'), 1.60 (2H, m, H-5'/H-6′), 1.20-1.82 (2H, m, H-7'), 1.81 (1H, m, H-1'),2.25 (3H, s, H8"), 3.22 (1H, d, H-4'), 8.24 (1H,dd H-3"/ H-5"), 7.83 (1H, dd, H-2"/H6"), 8.19 (1H, sl, H-4), 8.53 (1H, sl, NH, H-2); ^{13}C NMR (75.5 MHz, $CDCl_3$): δ 17.80 (C-8′),26.40 (C-10'), 23.50 (C9'), 23.40 (C-5′/C-6'), 35.00 (C-7'), 45.90 (C-2'), 48.00 (C-4'), 50.40 (C-1′), 66.90 (C-3'), 124.0 (C-3"/ C-5"), 126.80 (C-2"/C-6"), 13.4 (C-8"),142.0 (C-1"), 148.10 (C=N, C-7"), 143.7 (C-4"), 176.0 (C=S, C-3).

***p*-Chloroacetophenone (-)-camphene-based thiosemicarbazone (compound 20 in Fig 1)**

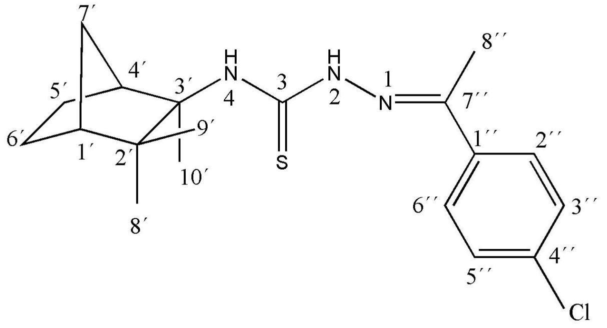

Yield 90%; EI-MS m/z 363.94 (M$^{+•}$); ^{1}H NMR (300 MHz, CDCl$_3$): δ$_H$ 1.27 (3H, s, H-10’), 1.05 (3H, s, H-9’), 1.75 (3H, s, H-8’), 1.60 (2H, m, H-5’/H-6′), 1.16-1.83 (2H, m, H-7’), 1.78 (1H, m, H-1’),2.22 (3H, s, H8”), 3.22 (1H, d, H-4’), 7.59 (1H,dd,2H, J=8.7, H-3”/ H-5”), 7.33 (1H, dd, J=8.7, H-2”/H6”), 8.19 (1H, s, H-4), 8.44 (1H, s, NH, H-2); ^{13}C NMR (75.5 MHz, CDCl$_3$): δ 17.80 (C-8′), 26.40 (C-10’), 23.50 (C9’), 23.30 (C-5′/C-6’), 34.9 (C-7’), 45.80 (C-2’), 47.90 (C-4’), 50.30 (C-1′), 66.60 (C-3’), 127.3 (C-3”/ C-5”), 128.90 (C-2”/C-6”), 13.4 (C-8”), 136.1 (C-1”), 143.9 (C=N, C-7”), 135.50 (C-4”), 175.9 (C=S, C-3).

***p*-Chloroacetophenone (-)-camphene-based thiosemicarbazone (compound 21 in Fig 1)**

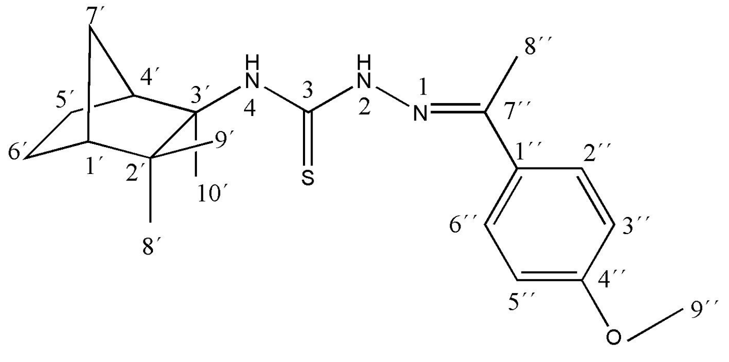

Yield 80%; EI-MS m/z 359.53 (M$^{+•}$); ^{1}H NMR (300 MHz, CDCl$_3$): δ$_H$ 1.31 (3H, s, H-10’), 1.07 (3H, s, H-9’), 1.75 (3H, s, H-8’), 1.60 (2H, m, H-5’/H-6′), 1.20-1.88 (2H, m, H-7’), 1.81 (1H,m, H-1’),2.22 (3H, s, H8”),3,85 (3H, s, H9′′); 3.25 (1H, m, H-4’), 6.90 (1H,dd, J=9.0, H-3”/ H-5”), 7.63 (1H, dd, J=9.0, H-2”/H6”), 8.24 (1H, s, H-4), 8.33 (1H, s, NH, H-2); ^{13}C NMR (75.5 MHz, CDCl$_3$): δ 18.0 (C-8′), 26.5 (C-10’), 23.6 (C9’), 23.40 (C-5′/C-6’), 35.0 (C-7’), 45.8 (C-2’), 48.1 (C-4’), 50.40 (C-1′), 66.6 (C-3’), 114.1 (C-3”/ C-5”), 127.6 (C-2”/C-6”), 13.4 (C-8”), 55,5 (C-9′′),130.0 (C-1”), 145.0 (C=N, C-7”), 160.9 (C-4”), 175.9 (C=S, C-3).

_p_-Methylacetophenone (-)-camphene-based thiosemicarbazone (compound 22 in Fig.1)

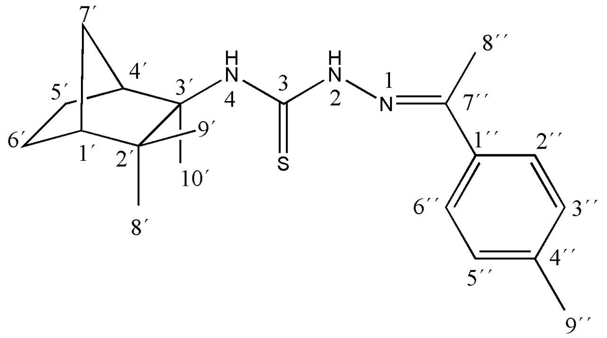

Yield 87%; EI-MS m/z 343.53 ($M^{+\bullet}$); ^{1}H NMR (300 MHz, $CDCl_3$): δ_H 1.29 (3H, s, H-10’), 1.05 (3H, s, H-9’), 1.74 (3H, s, H-8’), 1.60 (2H, m, H-5’/H-6´), 1.20-1.85 (2H, m, H-7’), 1.78 (1H, m, H-1’), 2.22 (3H, s, H8”), 2,37 (3H,s, H9´´), 3.24 (1H, m, H-4’), 7.18 (1H, dl, J=8.1, H-3”/ H-5”), 7.57 (1H, dd, J=8.4, H-2”/H6”), 8.26 (1H, s, H-4), 8.42 (1H, s, NH, H-2); ^{13}C NMR (75.5 MHz, $CDCl_3$): δ 17.9 (C-8´), 26.40 (C-10’), 23.50 (C9’), 23.30 (C-5´/C-6’), 34.9 (C-7’), 45.70 (C-2’), 48.0 (C-4’), 50.30 (C-1´), 66.5 (C-3’), 129.3 (C-3”/ C-5”), 126.0 (C-2”/C-6”), 13.4 (C-8”), 21,3 (C-9´´), 134.8 (C-1”), 145.3 (C=N, C-7”), 139.7 (C-4”), 175.9 (C=S, C-3).

_p_-Fluoracetophenone (-)-camphene-based thiosemicarbazone (compound 23 in Fig 1)

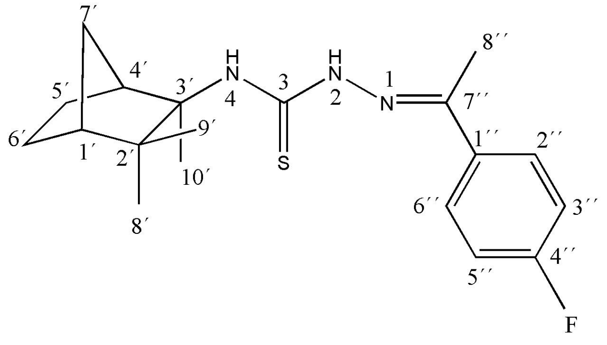

Yield 90%; EI-MS m/z 347.50 ($M^{+\bullet}$); ^{1}H NMR (300 MHz, $CDCl_3$): δ_H 1.29 (3H, s, H-10’), 1.07 (3H, s, H-9’), 1.75 (3H, s, H-8’), 1.60 (2H, m, H-5’/H-6´), 1.19-1.86 (2H, m, H-7’), 1.80 (1H, m, H-1’), 2.24 (3H, s, H8”), 3.24 (1H, m, H-4’), 7.08 (1H, dt, J=9.0, H-3”/ H-5”), 7.66 (1H, ddd, J=10.0, H-2”/H6”), 8.20 (1H, s, H-4), 8.36 (1H, s, NH, H-2); ^{13}C NMR (75.5 MHz, $CDCl_3$): δ 17.9 (C-8´), 26.5 (C-10’), 23.5 (C9’), 23.4 (C-5´/C-6’), 35.0 (C-7’), 45.8 (C-2’), 48.0 (C-4’), 50.4 (C-1´), 66.7 (C-3’), 115.8 (d, J=21.4, C-3”/ C-5”), 128.0 (d, J=8.2, C-2”/C-6”), 13.4 (C-8”), 133.9 (d, J=3.3, C-1”), 144.1 (C=N, C-7”), 163.7 (d, J=249, C-4”), 176.0 (C=S, C-3).

***p*-Hydroxyacetophenone (-)-camphene-based thiosemicarbazone (compound 24 in Fig 1)**

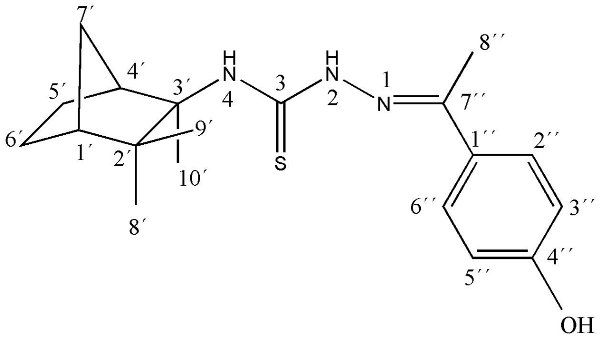

Yield 85%; EI-MS m/z 345.50 (M$^{+•}$); ^{1}H NMR (300 MHz, CDCl$_3$): δ$_H$ 1.27 (3H, s, H-10’), 1.05 (3H, s, H-9’), 1.73 (3H, s, H-8’), 1.60 (2H, m, H-5’/H-6′), 1.16-1.83 (2H, m, H-7’), 1.78 (1H, m, H-1’), 2.56 (3H, s, H8”), 3.22 (1H, m, H-4’), 6.92 (1H, dd, J=9.0, H-3”/ H-5”), 7.88 (1H, ddd, J=9.0, H-2”/H6”), 8.19 (1H, s, H-4), 8.44 (1H, s, NH, H-2); ^{13}C NMR (75.5 MHz, CDCl$_3$): δ 18.4 (C-8′), 26.6 (C-10’), 23.2 (C9’), 23.4 (C-5′/C-6’), 34.8 (C-7’), 46.0 (C-2’), 48.8 (C-4’), 50.5 (C-1′), 66.1 (C-3’), 115.6 (C-3”/ C-5”), 131.1 (C-2”/C-6”), 26.6 (C-8”), 127.8 (C-1”), 143.9 (C=N, C-7”), 160.0 (C-4”), 180.7 (C=S, C-3).

Thiophene-2-carboxyaldehyde (-)-camphene-based thiosemicarbazone (compound 25 in Fig 1)

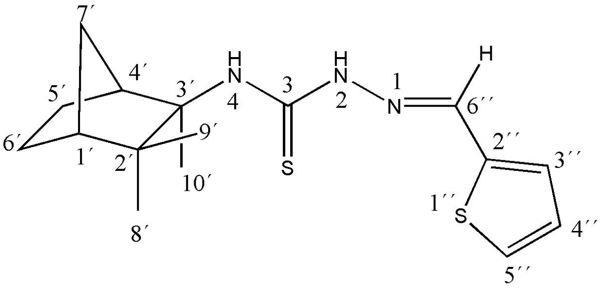

Yield 60%; EI-MS m/z 321.50 (M$^{+•}$); ^{1}H NMR (300 MHz, CDCl$_3$): δ$_H$ 1.31 (3H, s, H-10’), 1.06 (3H, s, H-9’), 1.72 (3H, s, H-8’), 1.60 (2H, m, H-5’/H-6′), 1.16-1.83 (2H, m, H-7’), 3.34 (1H, m, H-4’), 8.05 (1H, s, H6”), 7.33 (1H, dt,J=4.9, H-5”), 7.04 (1H, dd, J=4.9, H-4”), 7.23 (1H, dd, J=3.6, H-3”), 8.04 (1H, sl, H-4), 9.91 (1H, sl, NH, H-2); ^{13}C NMR (75.5 MHz, CDCl$_3$): δ 17.9 (C-8′), 26.3 (C-10’), 23.6 (C9’), 23.4 (C-5’), 23.6 (C-6’), 35.0 (C-7’), 45.8 (C-2’), 47.5 (C-4’), 50.3 (C-1′), 66.6 (C-3’), 128.19 (C-3”), 127.9 (C-4”), 130.2 (C-5”), 135.6 (C=N, C-6”), 139.0 (C-2”), 174.9 (C=S, C-3).

Furaldehyde (-)-camphene-based thiosemicarbazone (compound 26 in Fig 1)

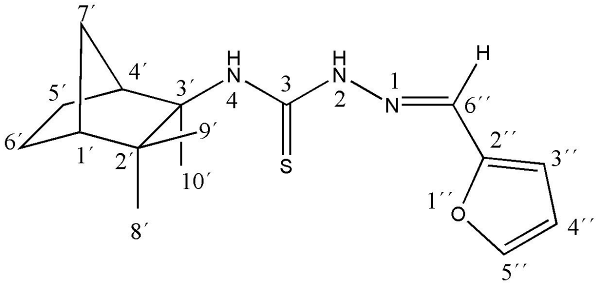

Yield 40%; EI-MS m/z 305.43 ($M^{+\bullet}$); ^{1}H NMR (300 MHz, $CDCl_3$): δ_H 1.31 (3H, s, H-10'), 1.06 (3H, s, H-9'), 1.72 (3H, s, H-8'),1.60 (2H, m, H-5'/H-6′), 1.16-1.83 (2H, m, H-7'), 1.80 (1H, m, H-1'), 3.34 (1H, d, J=3.3, H-4'), 7.75 (1H, s, H6"), 7.48 (1H, m, H-5"), 6.46 (1H, dd,J=3.6, H-4"), 6.65 (1H, d, J=3.6, H-3"), 8.67 (1H, sl, H-1"), 8.04 (1H, s, H-4), 9.91 (1H, s, NH, H-2); ^{13}C NMR (75.5 MHz, $CDCl_3$): δ 18.0 (C-8′), 26.3 (C-10'), 23.5 (C9'), 23.4 (C-5'/C-6'), 35.0 (C-7'), 45.9 (C-2'), 48.1 (C-4'), 50.4 (C-1′), 66.6 (C-3'), 113.0 (C-3"), 112.1 (C-4"), 144.6 (C-5"), 130.7 (C=N, C-6"), 149.4 (C-2"), 175.2 (C=S, C-3).

1H-pyrrole-2-carboxyaldehyde (-)-camphene-based thiosemicarbazone (compound 27 in Fig 1)

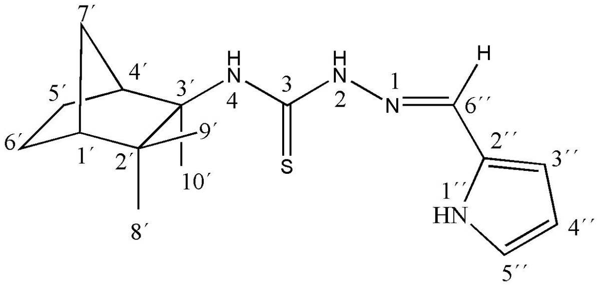

Yield 40%; EI-MS m/z 304.45 ($M^{+\bullet}$); ^{1}H NMR (300 MHz, $CDCl_3$): δ_H 1.31 (3H, s, H-10'), 1.08 (3H, s, H-9'), 1.75 (3H, s, H-8'), 1.60 (2H, m, H-5'/H-6′), 1.19-1.86 (2H, m, H-7'), 3.16 (1H, m, H-4'), 8.05 (1H, s, H6"), 6.50 (1H, m, H-5"), 6.27 (1H, dt, J=6.0, H-4"), 6.93 (1H, dd, J=3.9, H-3"), 7.72 (1H, sl, H-4), 8.90 (1H, sl, NH, H-2); ^{13}C NMR (75.5 MHz, $CDCl_3$): δ 18.2 (C-8′), 26.5 (C-10'), 23.5 (C9'), 23.3 (C-5'), 23.5 (C-6'), 35.0 (C-7'), 46.0 (C-2'), 48.5 (C-4'), 50.5 (C-1′), 66.6 (C-3'), 114.9 (C-3"), 110.8 (C-4"), 122.1 (C-5"), 133.5 (C=N, C-6"), 126.8 (C-2"), 175.2 (C=S, C-3).

Benzophenone (-)-camphene-based thiosemicarbazone (compound 28 in Fig 1)

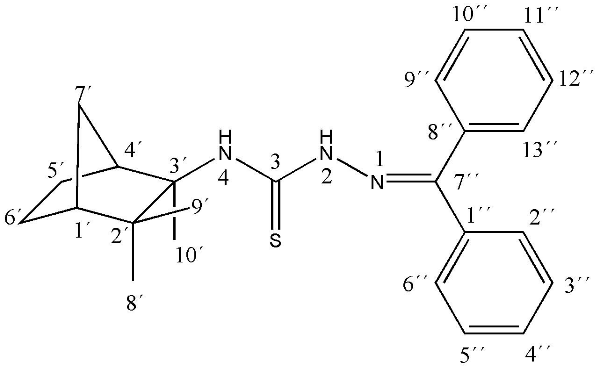

Yield 97%; EI-MS m/z 391.57 ($M^{+\bullet}$); ^{1}H NMR (300 MHz, $CDCl_3$): δ_H 1.36 (3H, s, H-10’), 1.08 (3H, s, H-9’), 1.75 (3H, s, H-8’), 1.60 (2H, m, H-5’/H-6′), 1.20-1.83 (2H, m, H-7’), 3.28 (1H, m, H-4’), 7.33 (1H, s, H6”), 7.27 (1H,H3”/ H-5”), 7.33 (1H, m, H2”/H6”),7.47 (1H, m, H9”/H13”), 7.54 (1H, m, H10”/H12”), 7.55 (1H, m, H-4”/H11”), 8.33 (1H, sl, H-4), 8.39 (1H, sl, NH, H-2); ^{13}C NMR (75.5 MHz, $CDCl_3$): δ 18.0 (C-8′), 26.6 (C-10’), 23.6 (C9’), 23.4 (C-5’/C6′), 35.1 (C-7’), 45.9 (C-2’), 48.0 (C-4’), 50.3 (C-1′), 66.7 (C-3’), 128.7 (C-3”), 129.9 (C-4”), 128.7 (C-5”), 128.6 (C-6”), 148.1(C=N, C-7”), 137.0 (C-8”), 127.5 (C-9”), 131.4 (C-1”), 128.6 (C-2”), 138.7 (C-10”), 130.3 (C-11”), 130.0 (C-12”),127.5 (C-13”), 175.6 (C=S, C-3).

Cinnamaldehyde (-)-camphene-based thiosemicarbazone (compound 29 in Fig 1)

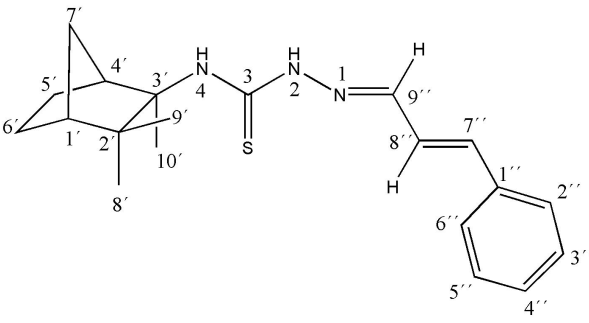

Yield 95%; EI-MS m/z 341.51 ($M^{+\bullet}$); ^{1}H NMR (300 MHz, $CDCl_3$): δ_H 1.28 (3H, s, H-10’), 1.08 (3H, s, H-9’), 1.75 (3H, s, H-8’),1.60 (2H, m, H-5’/H-6′), 1.18-1.88 (2H, m, H-7’), 1.80 (1H, m, H-1’), 3.06 (1H, m, H-4’), 7.62 (1H, d, J=8.7, H9”), 7.47 (1H, dd, J= 7.8, H2”/H6”), 7.37 (1H, m,H-3”/H-5”), 7.34 (1H, m, H-4”), 6.90 (1H, d, J=15.9, H-7”), 7.86 (1H, sl, H-4), 9.30 (1H, sl, NH, H-2); ^{13}C NMR (75.5 MHz, $CDCl_3$): δ 18.2 (C-8′), 26.5 (C-10’), 23.6 (C9’), 23.5 (C-5’), 23.3 (C-6′), 35.0 (C-7’), 46.1 (C-2’), 48.8 (C-4’), 50.5 (C-1′), 66.7 (C-3’), 127.2 (C-3”), 129.2 (C-4”), 127.2 (C-5”), 129.0 (C-6”), 124.7 (C=N, C-7”), 129.0 (C-2”), 124.7 (C-8”), 136.0 (C-1”), 175.4 (C=S, C-3).

Menthone (-)-camphene-based thiosemicarbazone (compound 30 in Fig 1)

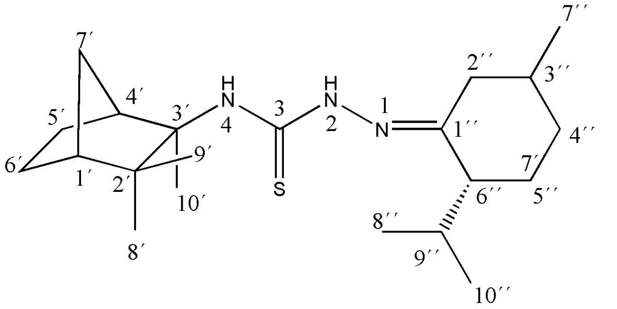

Yield 40%; EI-MS m/z 363.60 ($M^{+•}$); ^{1}H NMR (300 MHz, $CDCl_3$): δ_H 1.23 (3H, s, H-10’), 1.06 (3H, s, H-9’), 1.75 (3H, s, H-8’), 1.60 (2H, m, H-5’/H-6′), 1.18-1.88 (2H, m, H-7’), 1.80 (1H, m, H-1’), 3.06 (1H, m, H-4’), 2.62 (2H, m, H2”), 2.22 (1H, m, H3”), 1.64 (2H, m, H4”), 196.0 (2H, m, H5”), 160.0 (1H, dd, J=9.0, H6”), 0.89 (3H, d, J=6.9, H-7”), 0.94 (3H, d, J=6.9, H-8”), 1.86 (1H,m, H9”), 0.99 (3H,d,J=6.9,H-10”), (1H, m, H-4”), 8.04 (1H, sl, H-4), 8.26 (1H, sl, NH, H-2); ^{13}C NMR (75.5 MHz, $CDCl_3$): δ 18.3 (C-8′), 26.4 (C-10’), 23.5 (C9’), 23.5 (C-5’), 23.3 (C-6′), 35.0 (C-7’), 45.9 (C-2’), 49.1 (C-4’), 50.5 (C-1′), 66.5 (C-3’), 26.5 (C-3”), 33.9 (C-4”), 27.7 (C-5”), 33.6 (C-6”), 18.9 (C-7”), 34.8 (C-2”), 22.0 (C-8”), 22.2 (C-10′′), 154.0 (C-1”), 175.5 (C=S, C-3).

Ethylpyruvate (-)-camphene-based thiosemicarbazone (compound 31 in Fig 1)

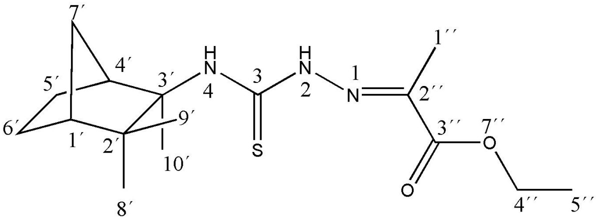

Yield 80%; EI-MS m/z 325.47 ($M^{+•}$); ^{1}H NMR (300 MHz, $CDCl_3$): δ_H 1.25 (3H, s, H-10’), 1.04 (3H, s, H-9’), 1.69 (3H, s, H-8’), 1.60 (2H, m, H-5’/H-6′), 1.17-1.88 (2H, m, H-7’), 1.78 (1H, m, H-1’), 3.31 (1H, m, H-4’), 2.07 (3H, s, H1”), 4.25 (2H, q,J=7.0, H4”), 1.31 (3H, t,J=7.0, H5”), 8.41 (1H, sl, H-4), 8.41 (1H, s, NH, H-2); ^{13}C NMR (75.5 MHz, $CDCl_3$): δ 17.4 (C-8′), 26.2 (C-10’), 23.6 (C9’), 23.5 (C-5’), 23.3 (C-6′), 35.0 (C-7’), 45.8 (C-2’), 50.3 (C-1′/C-4’), 66.9 (C-3’), 164.5 (C-3”), 61.8 (C-4”), 14.2 (C-5”), 135.6 (C-2”), 22.0 (C-8”), 11.4 (C-1”), 17575 (C=S, C-3).

S1.2 Spectral Data

p-M**ethyl-benzaldehyde (-)-camphene- based thiosemicarbazone (compound 5 in Fig 1)**

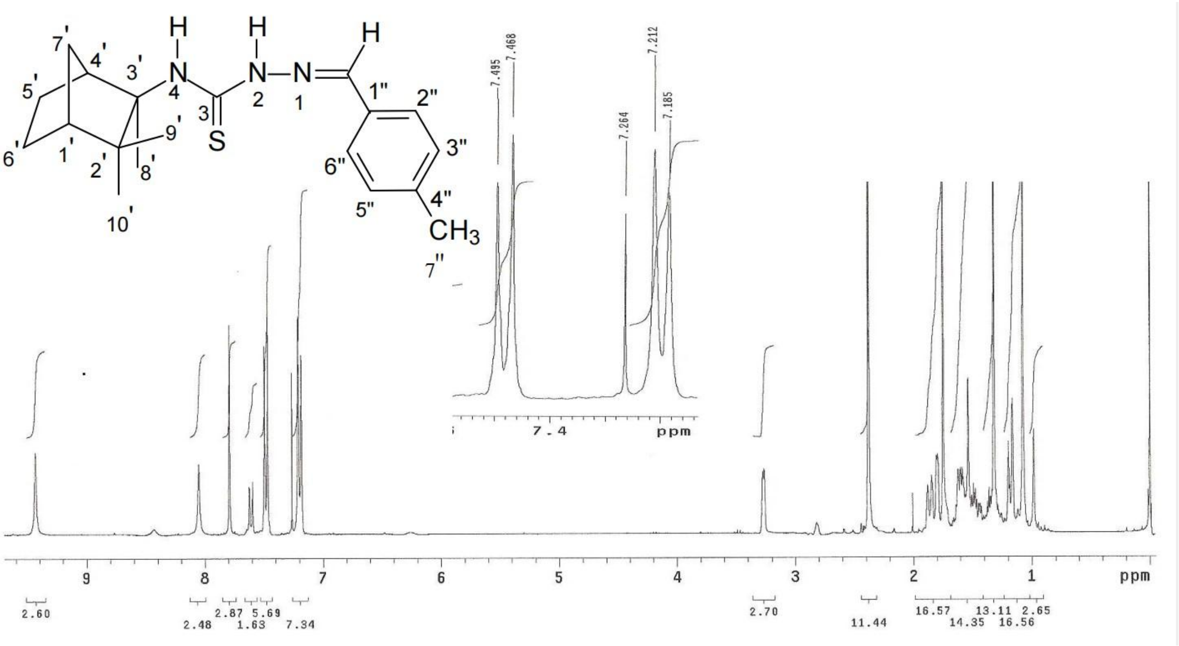

^{1}H NMR spectrum (300.06 MHz, $CDCl_3$) of compound **5**.

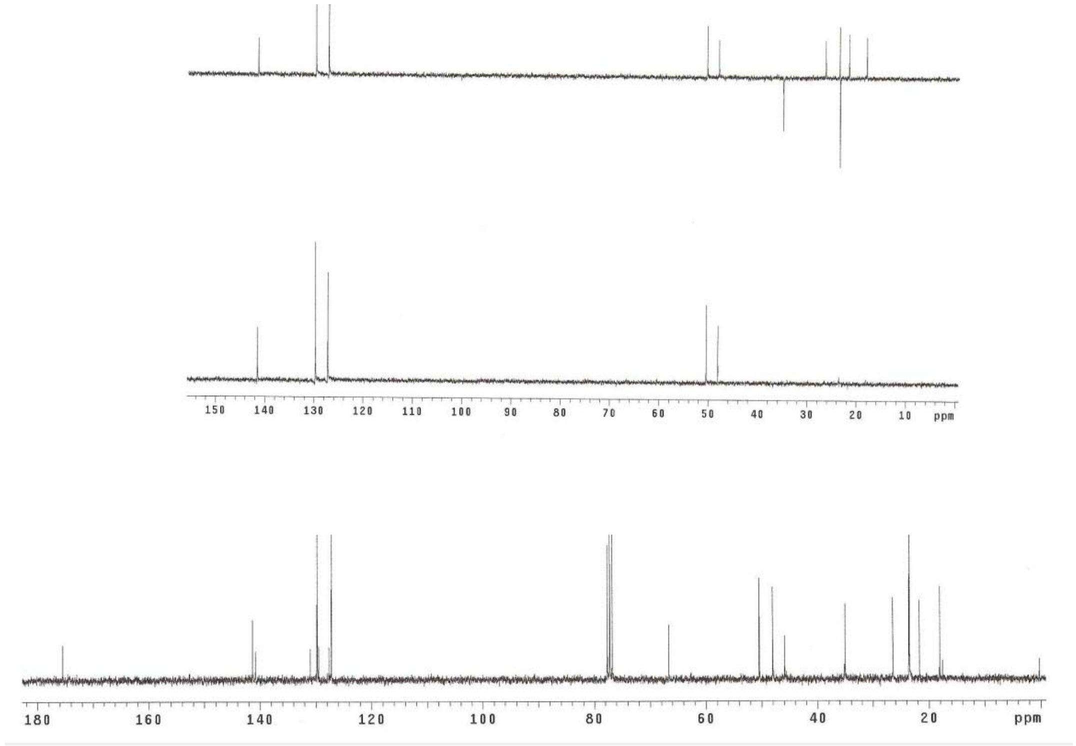

^{13}C/DEPT NMR spectrum (75.45 MHz, $CDCl_3$) of compound **5**.

***m*-Chloro-benzaldehyde (-)-camphene-based thiosemicarbazone (compound 8 in Fig 1)**

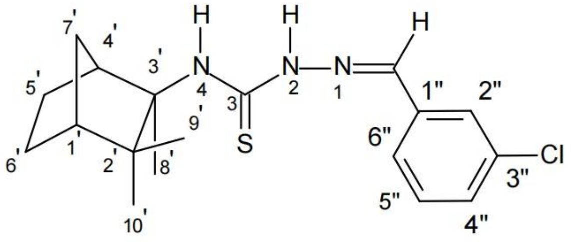

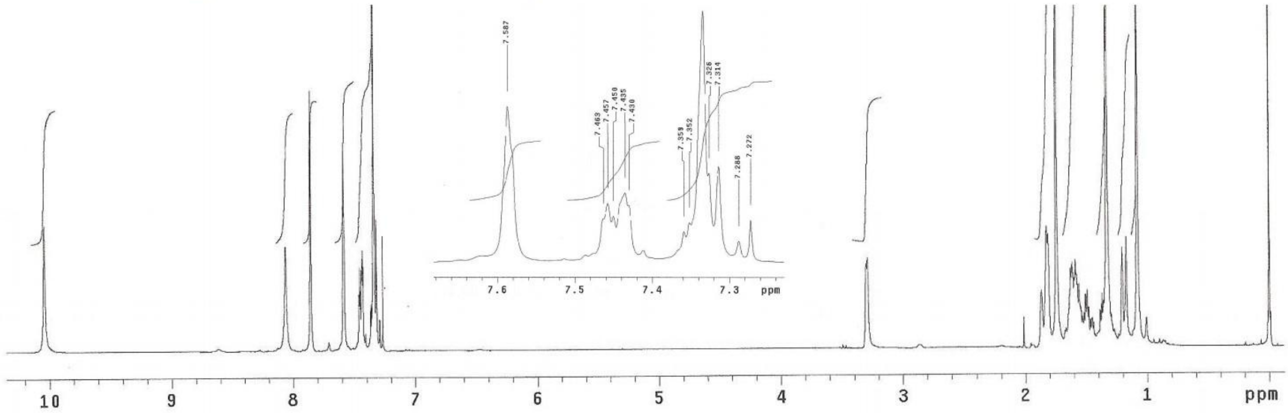

^{1}H NMR spectrum (300.06 MHz, $CDCl_3$) of compound **8.**

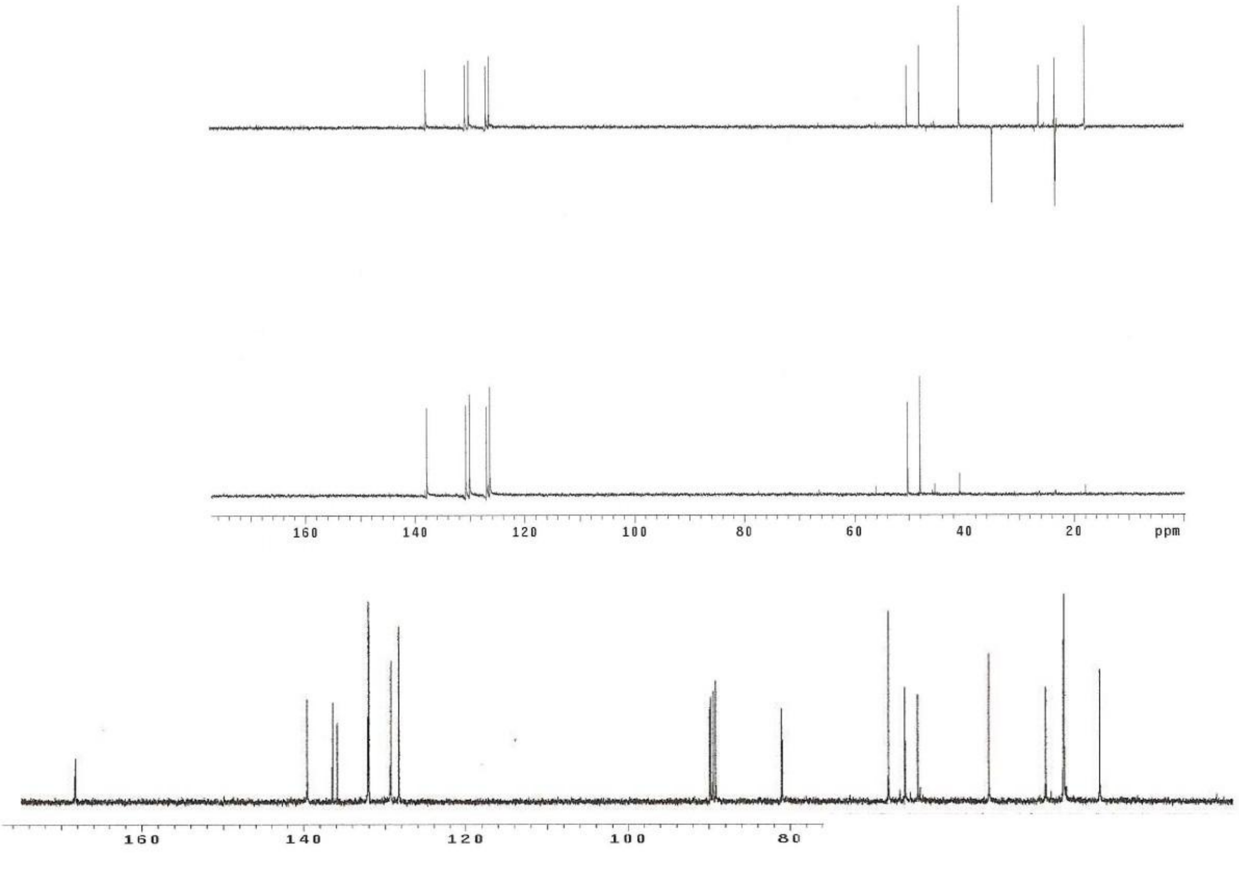

^{13}C/DEPT NMR spectrum (75.45 MHz, $CDCl_3$) of compound **8**.

***o*-Nitro-benzaldehyde (-)-camphene-based thiosemicarbazone (compound 10 in Fig 1)**

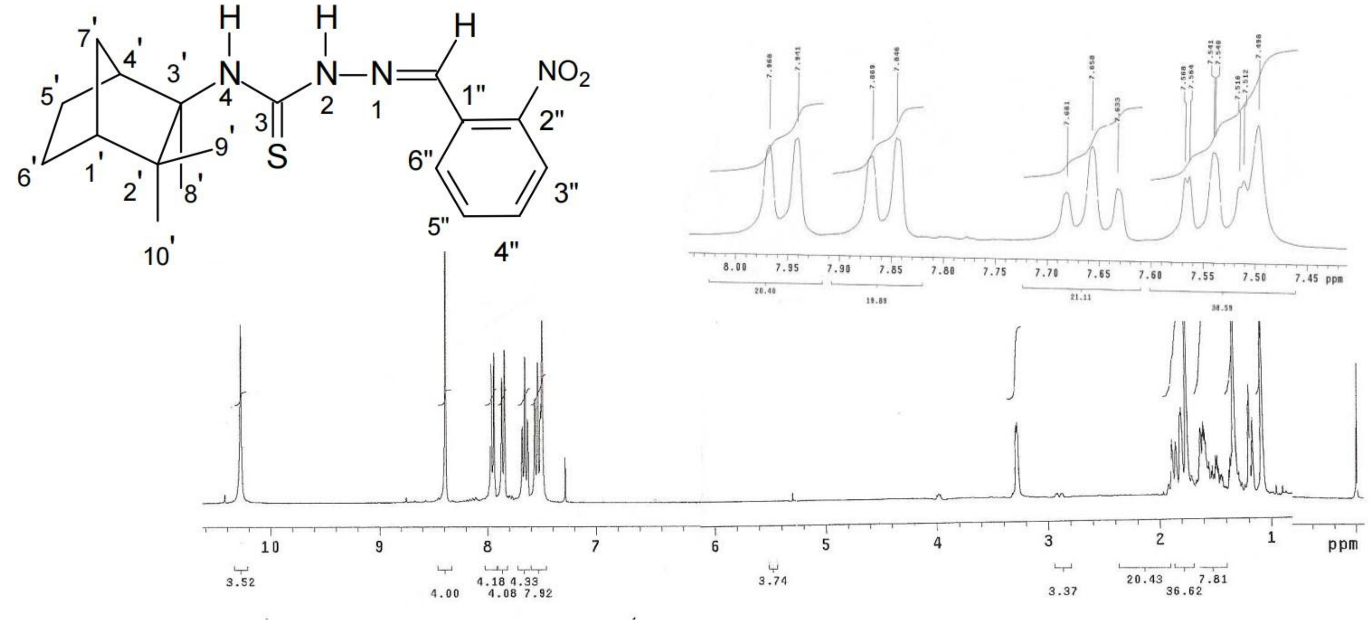

^{1}H NMR spectrum (300.06 MHz, $CDCl_3$) of compound **10**.

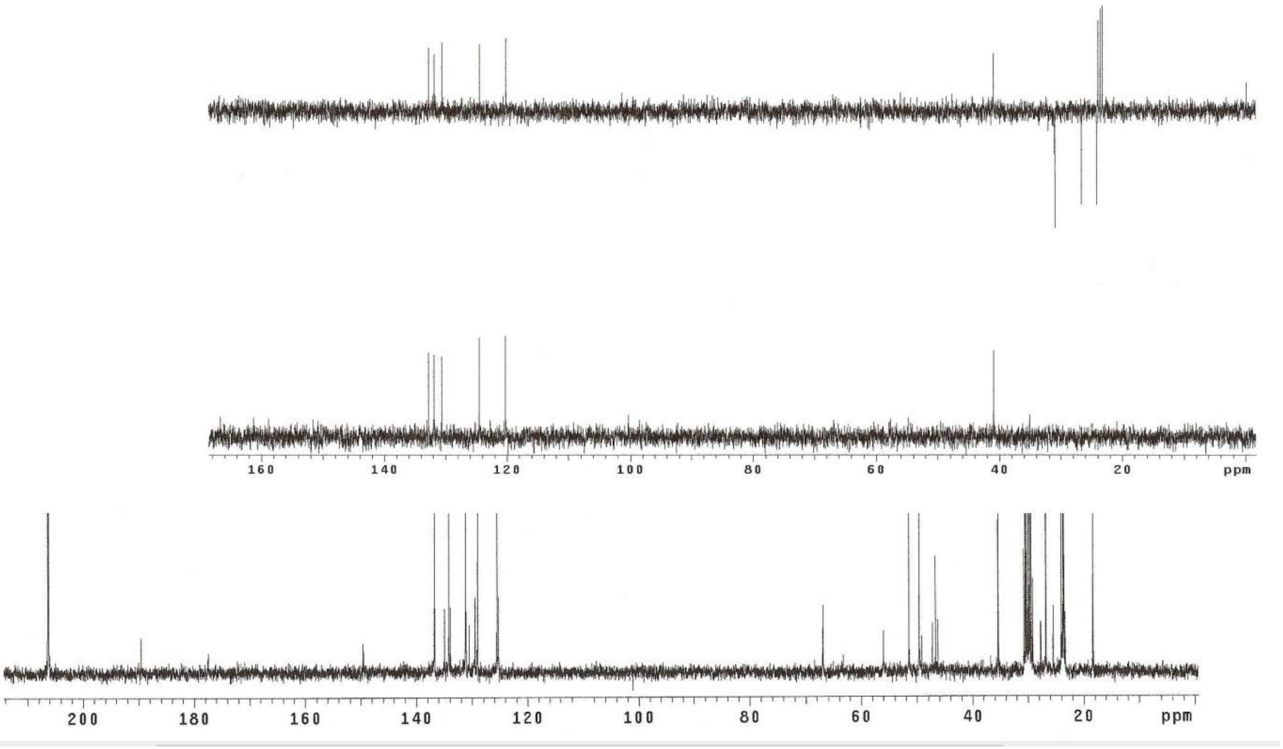

^{13}C/DEPT NMR spectrum (75.45 MHz, $CDCl_3$) of compound **10**.

_m_-Nitro-benzaldehyde (-)-camphene-based thiosemicarbazone (compound 11 in Fig 1)

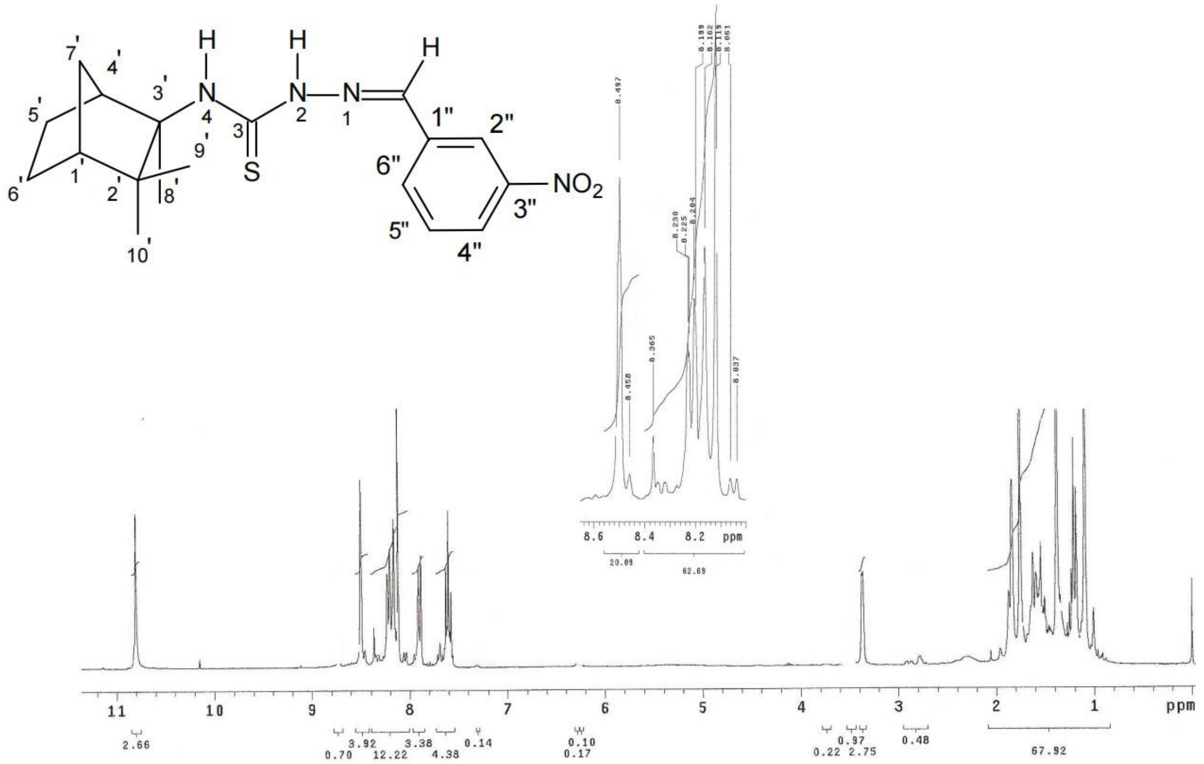

^{1}H NMR spectrum (300.06 MHz, $CDCl_3$) of compound **11**.

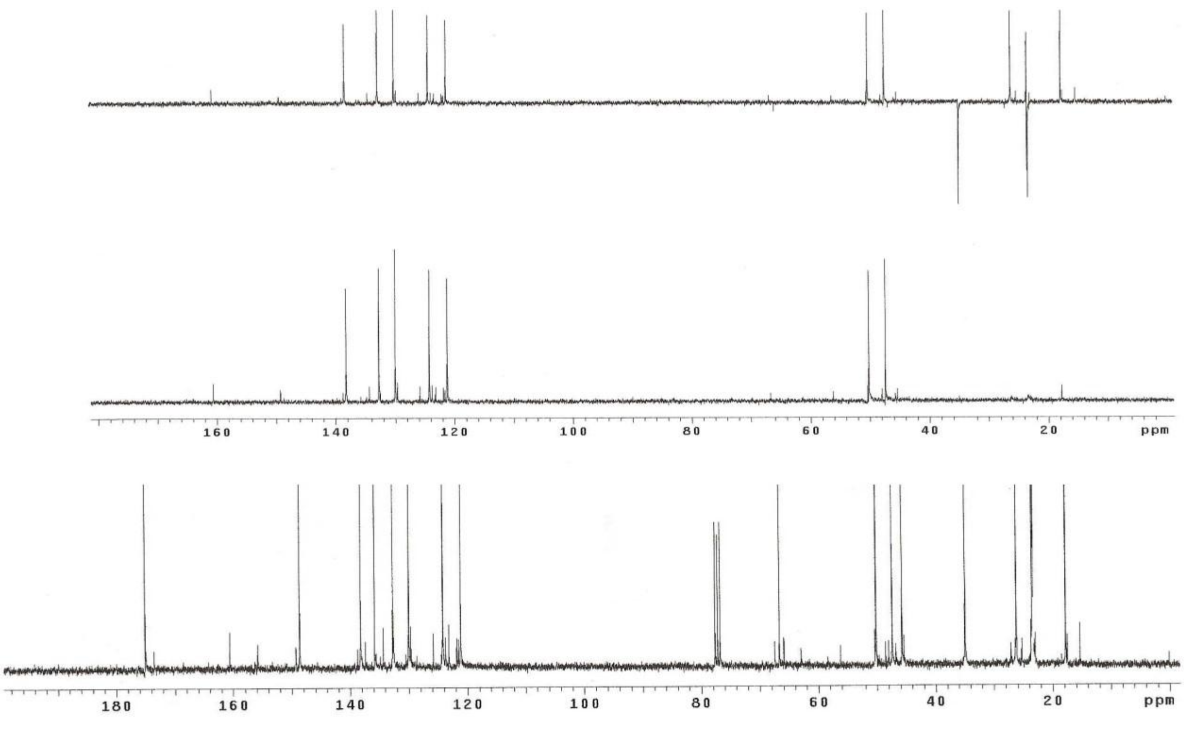

^{13}C/DEPT NMR spectrum (75.45 MHz, $CDCl_3$) of compound **11**.

m-Methoxy-p-hydroxybenzaldehyde (-)-camphene-based thiosemicarbazone (compound 15 in Fig 1)

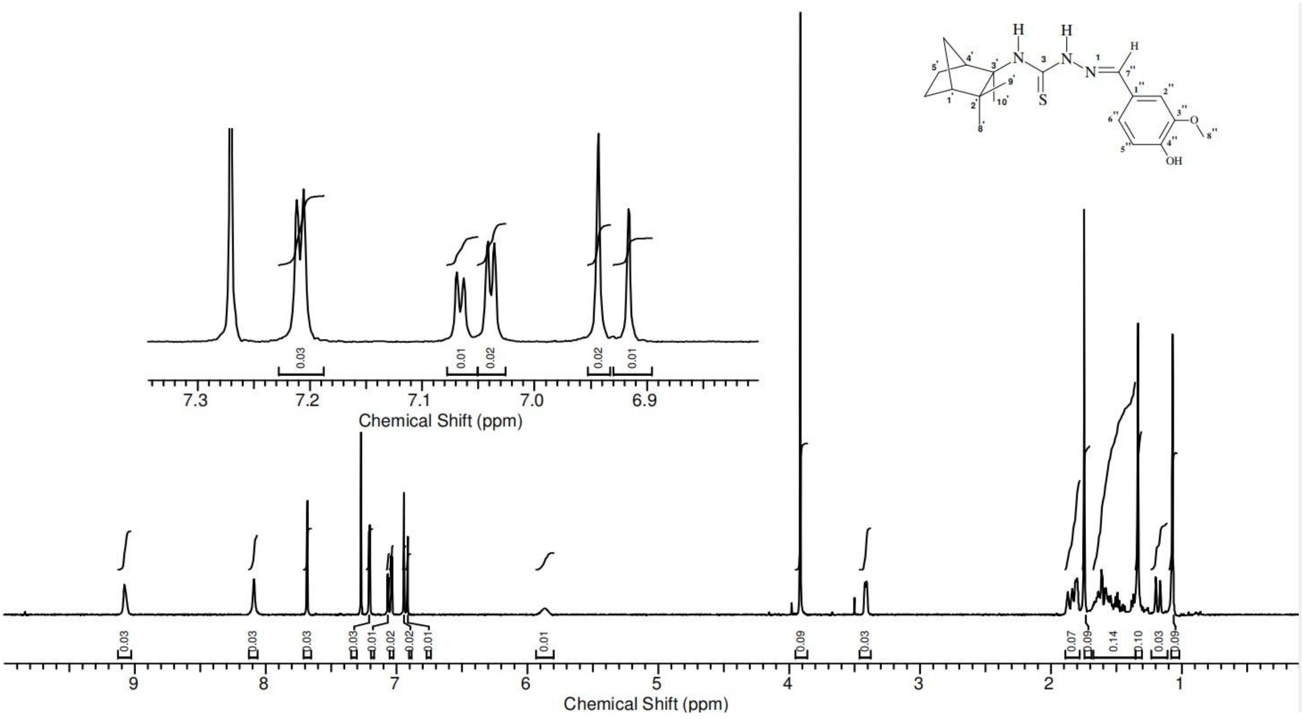

^{1}H NMR spectrum (300.06 MHz, $CDCl_3$) of compound **15**.

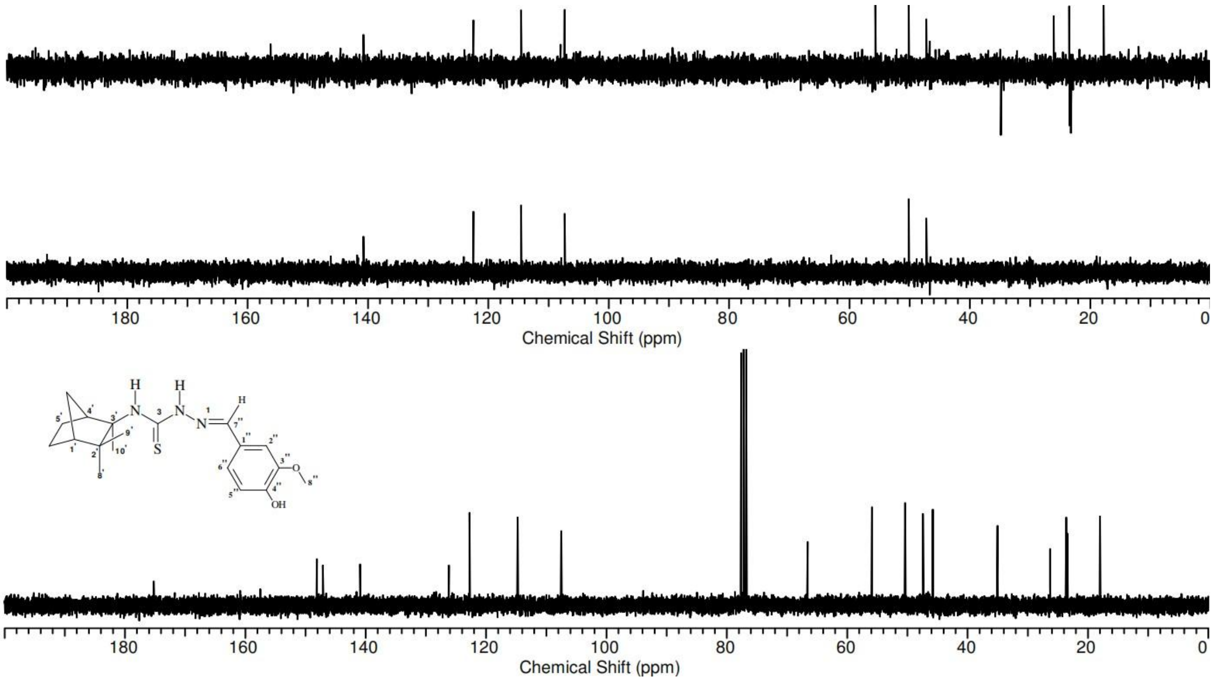

^{13}C(a)NMR/DEPT 90°(b) and DEPT 135°(c) spectra (75.45 MHz, $CDCl_3$) of compound **15**.

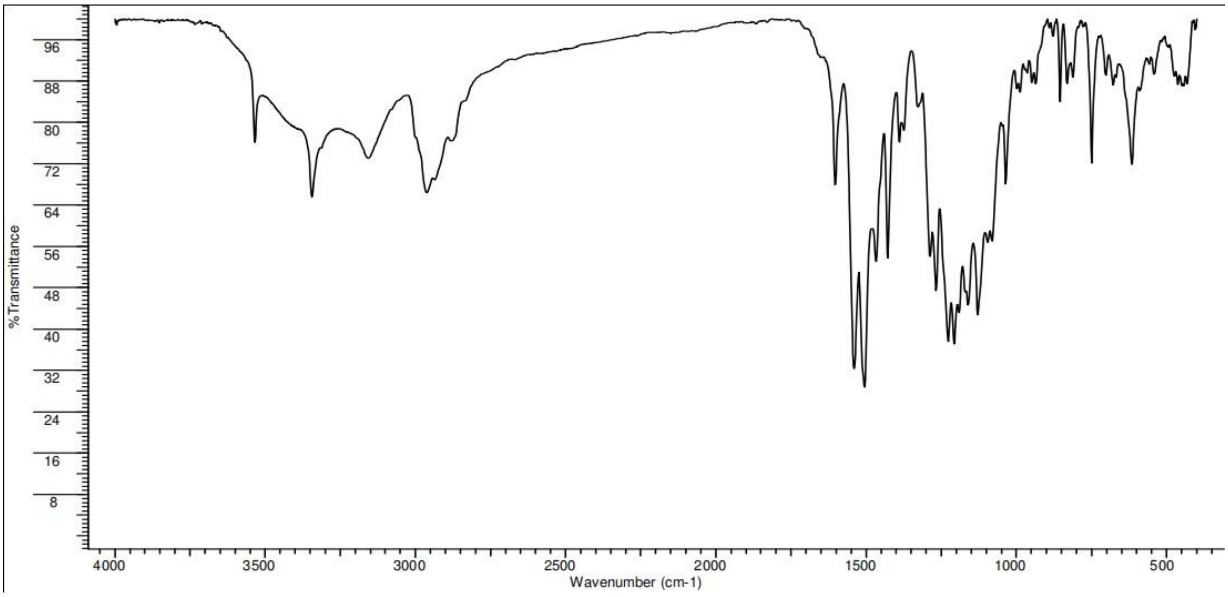

Infrared spectrum (KBr pellet) of compound **15**.

Acetophenone (-)-camphene-based thiosemicarbazone (compound 18 in Fig 1)

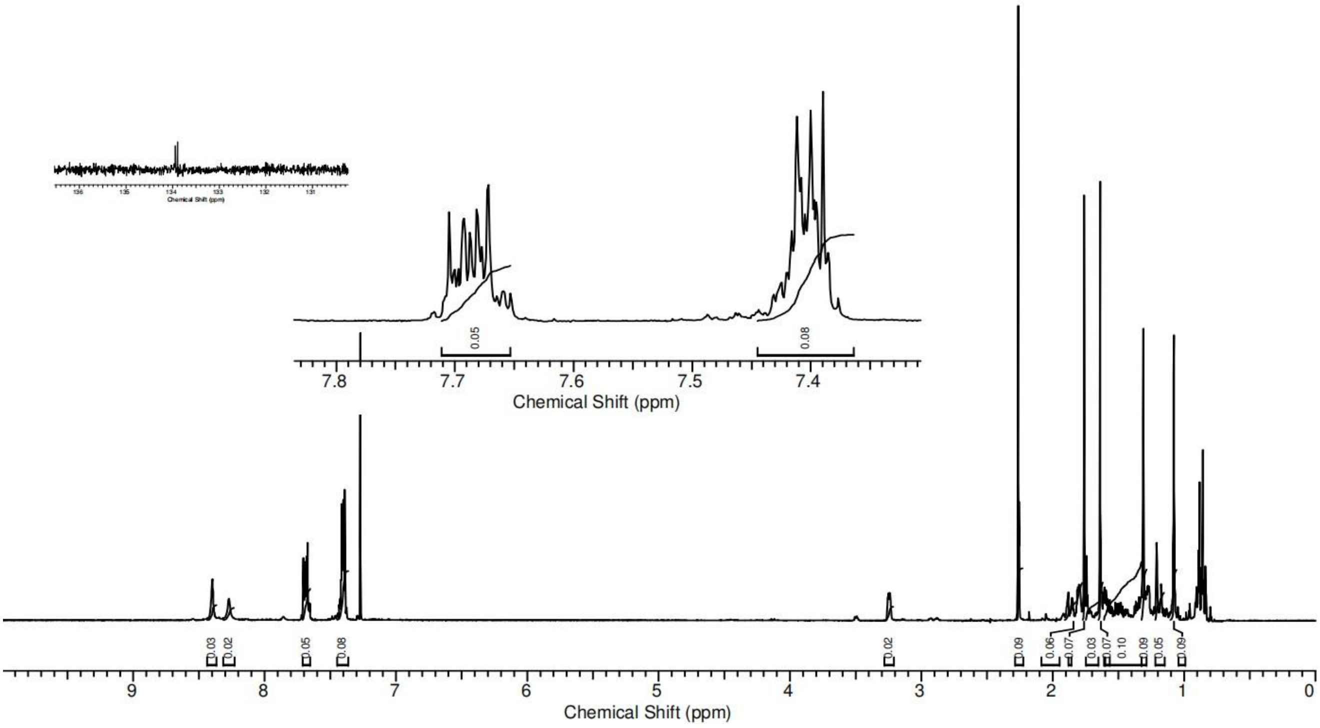

^{1}H NMR spectrum (300.06 MHz, $CDCl_3$) of compound **18**.

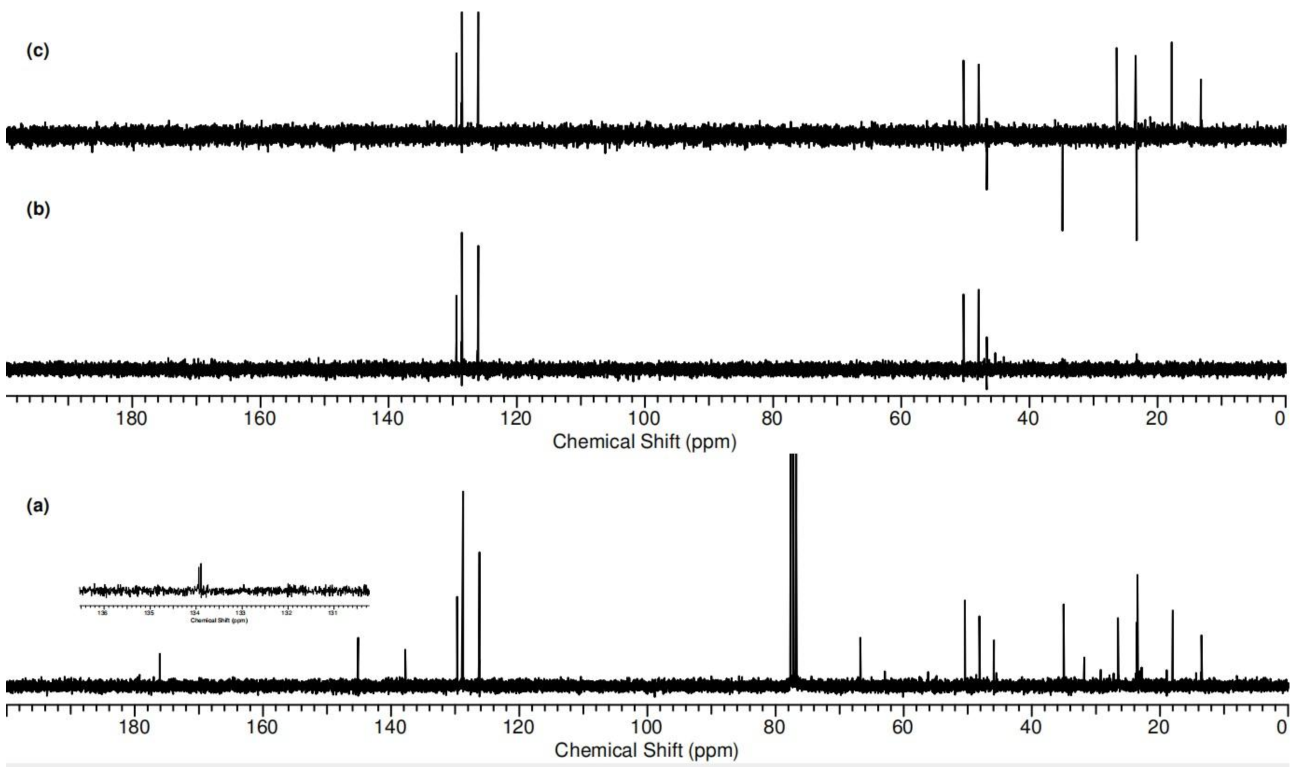

^{13}C(a)NMR/DEPT 90°(b) and DEPT 135°(c) spectra (75.45 MHz, $CDCl_3$) of compound 18.

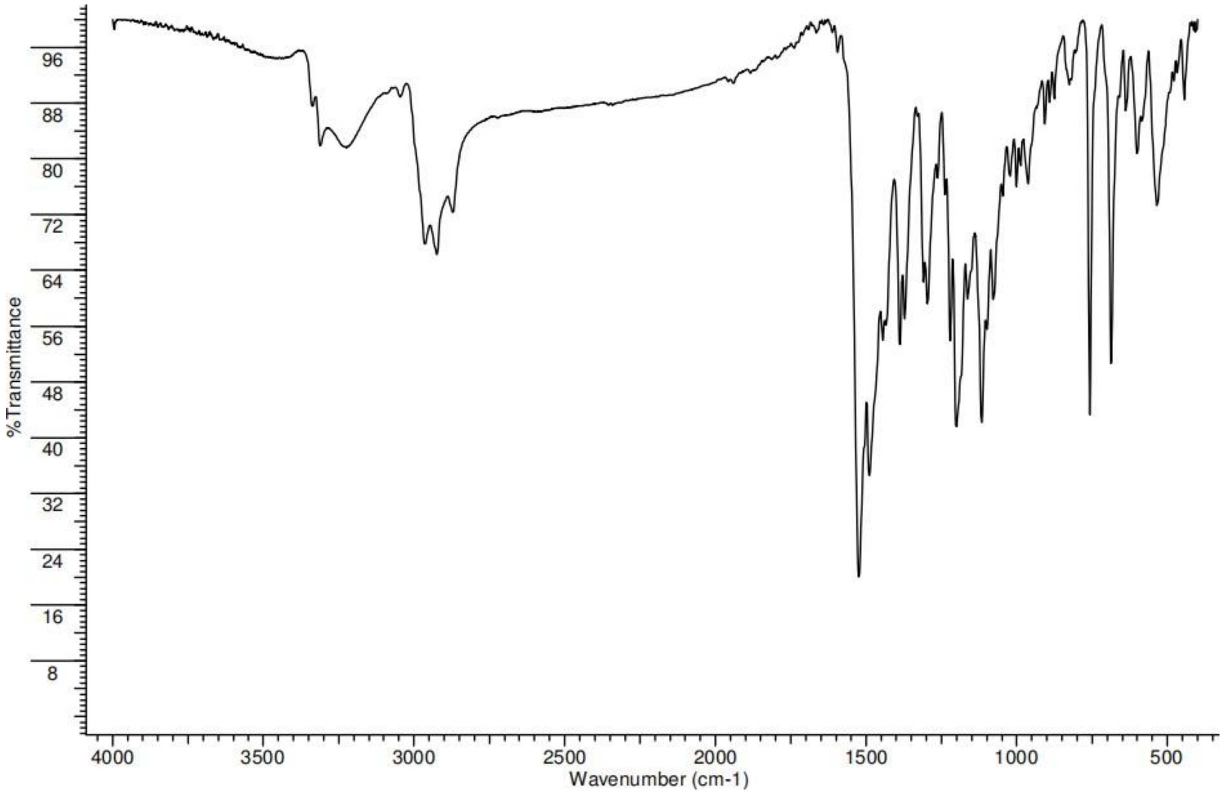

Infrared spectrum (KBr pellet) of compound **18**.

p-Nitroacetophenone (-)-camphene-based thiosemicarbazone (compound 19 in Fig 1)

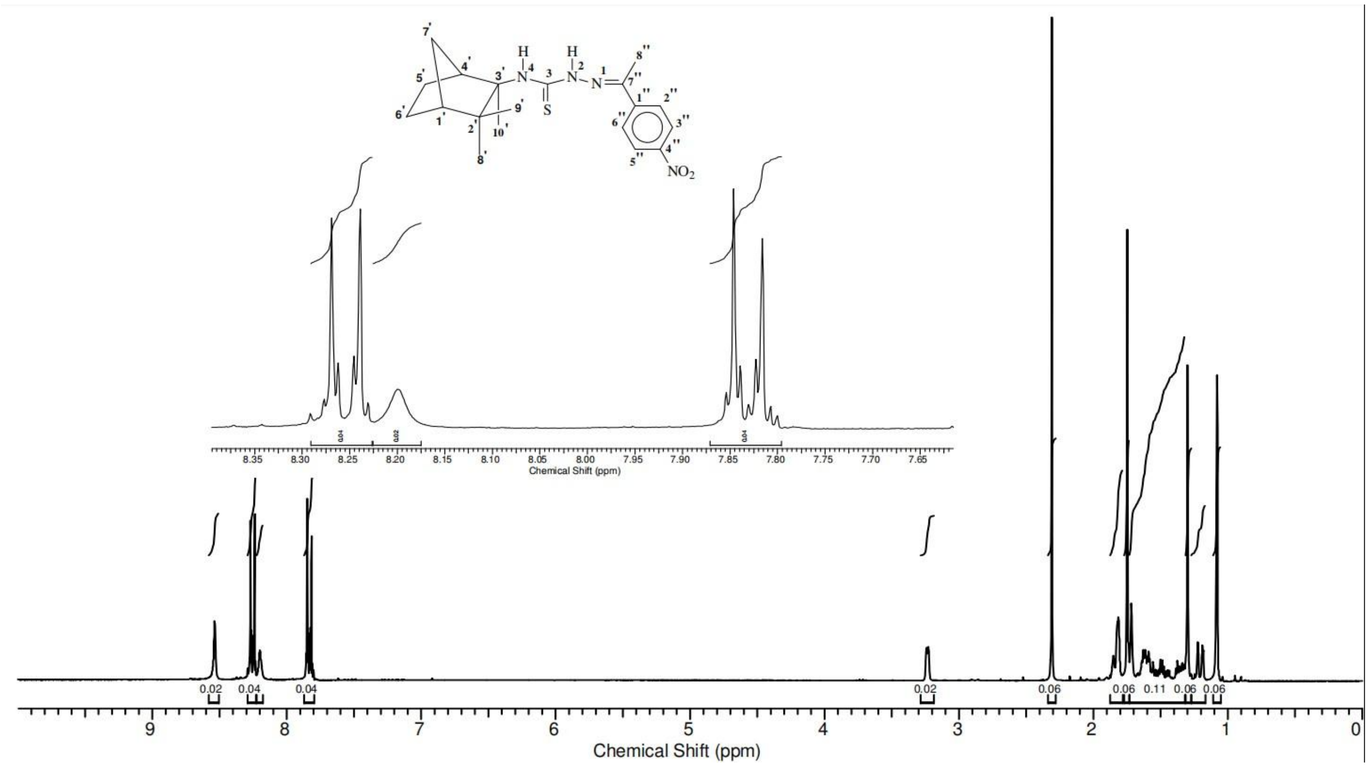

^{1}H NMR spectrum (300.06 MHz, $CDCl_3$) of compound **19**.

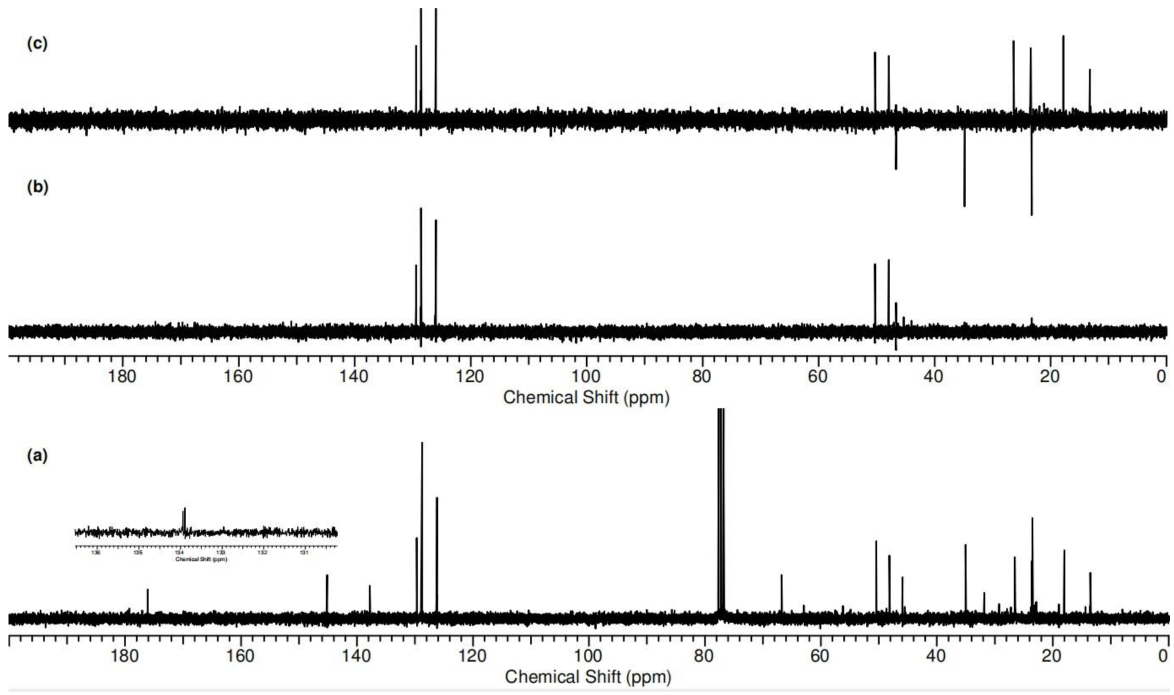

^{13}C(a)NMR/DEPT 90°(b) and DEPT 135°(c) spectra (75.45 MHz, $CDCl_3$) of compound **19**.

_p_-Chloroacetophenone (-)-camphene-based thiosemicarbazone (compound 20 in Fig 1)

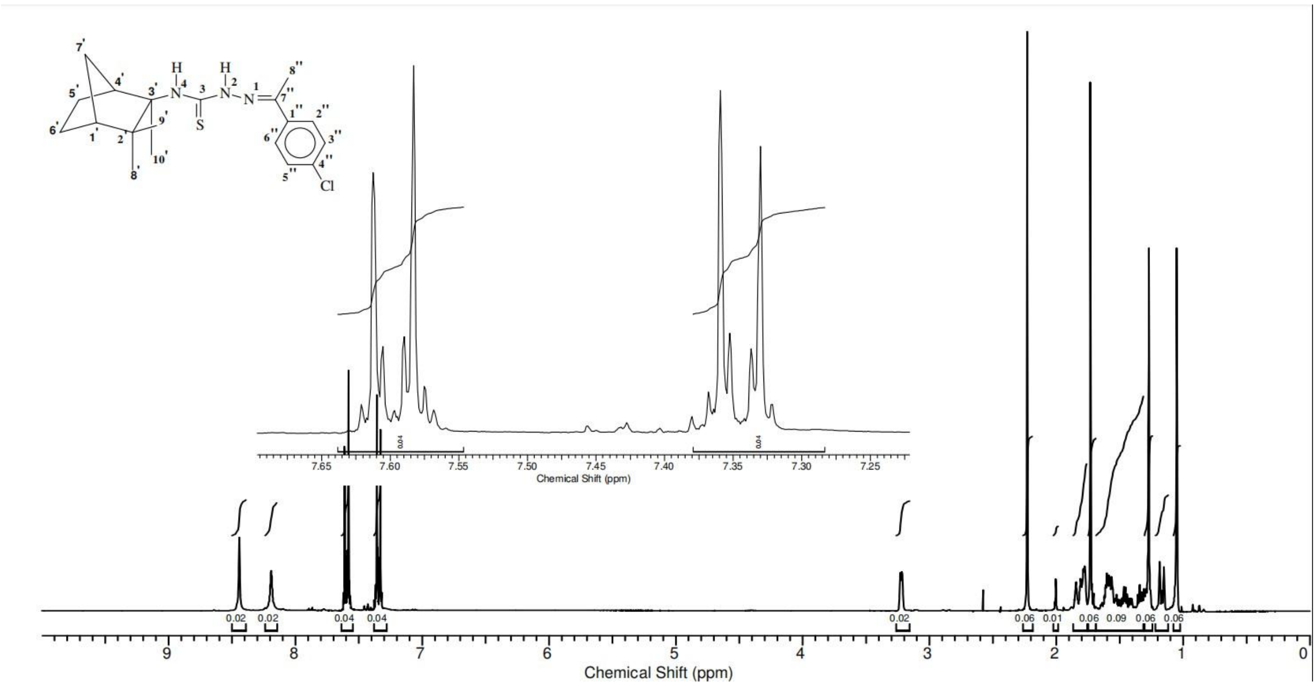

^{1}H NMR spectrum (300.06 MHz, $CDCl_3$) of compound **20**.

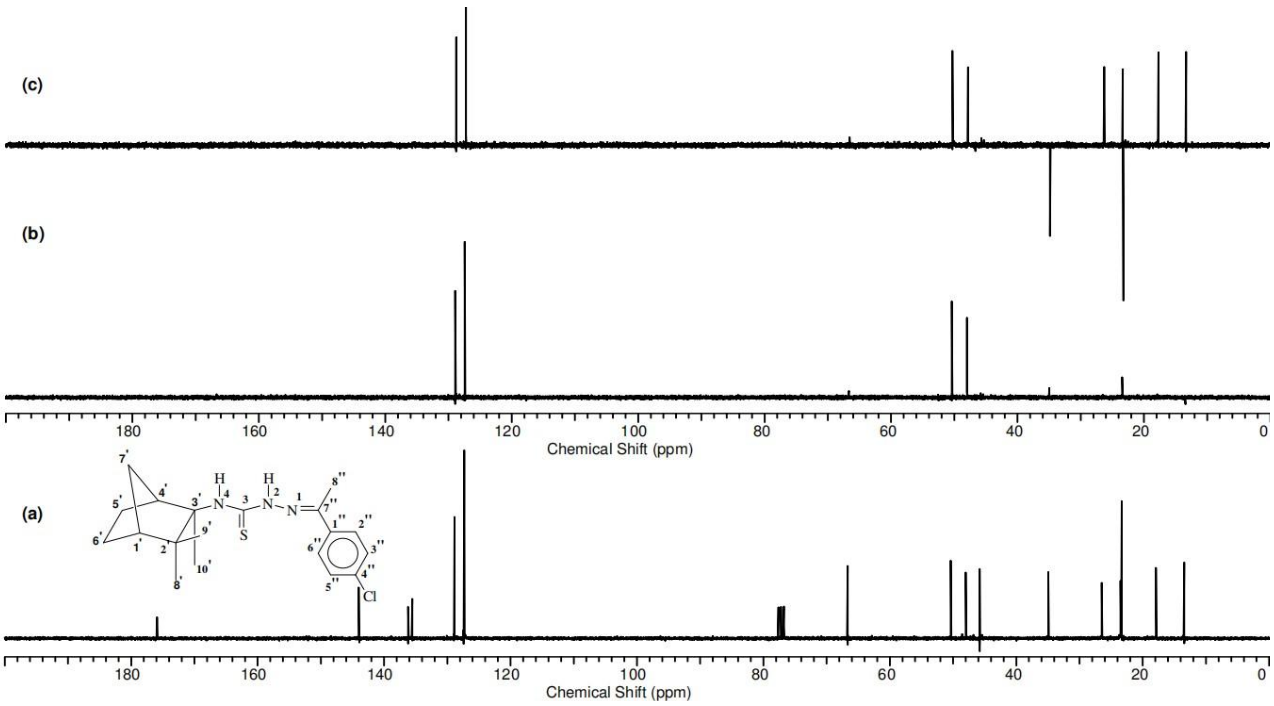

^{13}C(a)NMR/DEPT 90°(b) and DEPT 135°(c) spectra (75.45 MHz, $CDCl_3$) of compound **20**.

***p*-Methoxyacetophenone (-)-camphene-based thiosemicarbazone (compound 21 in Fig 1)**

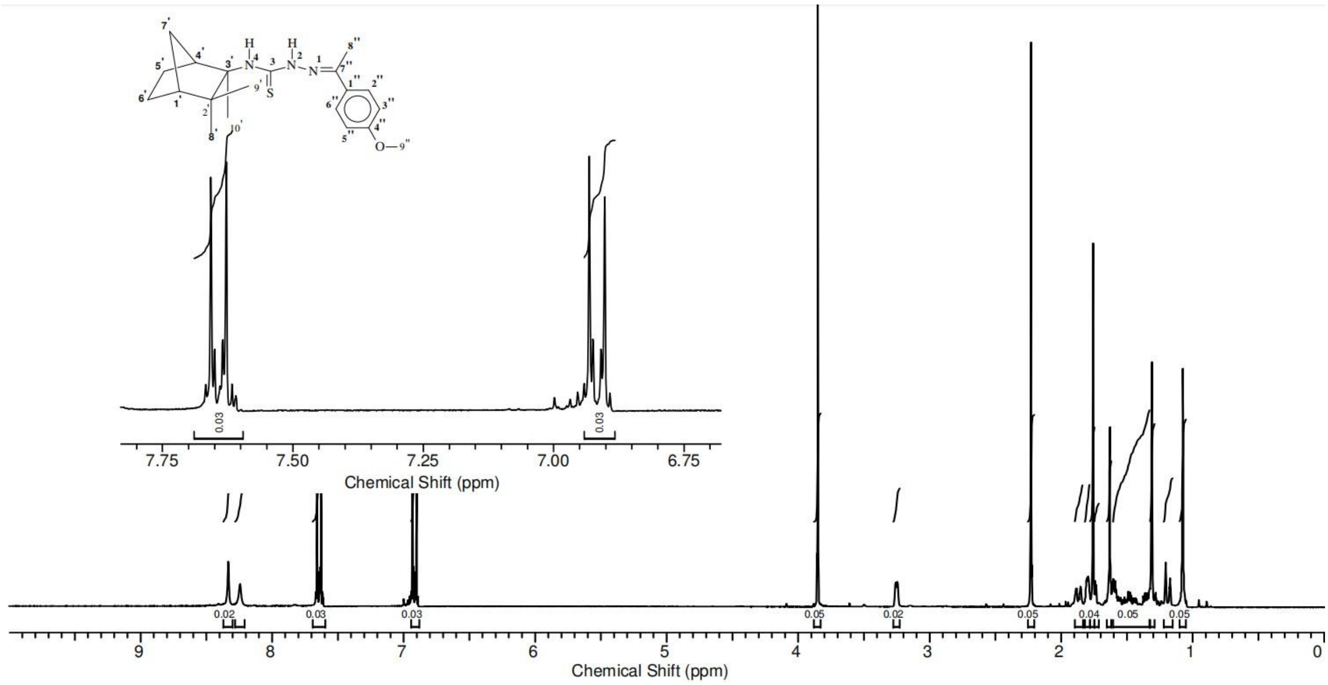

^{1}H NMR spectrum (300.06 MHz, $CDCl_3$) of compound **21**.

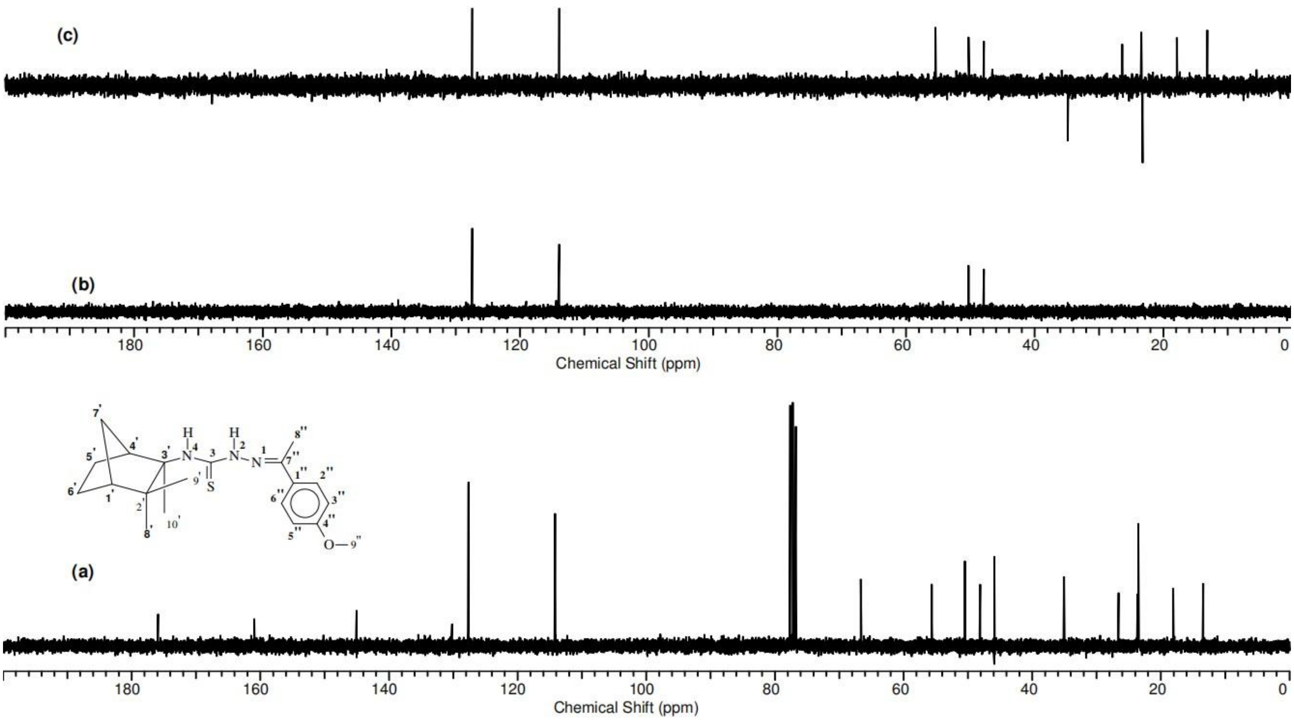

^{3}C(a)NMR/DEPT 90°(b) and DEPT 135°(c) spectra (75.45 MHz, $CDCl_3$) of compound **21**.

***p*-Methylacetophenone (-)-camphene-based thiosemicarbazone (compound 22 in Fig.1)**

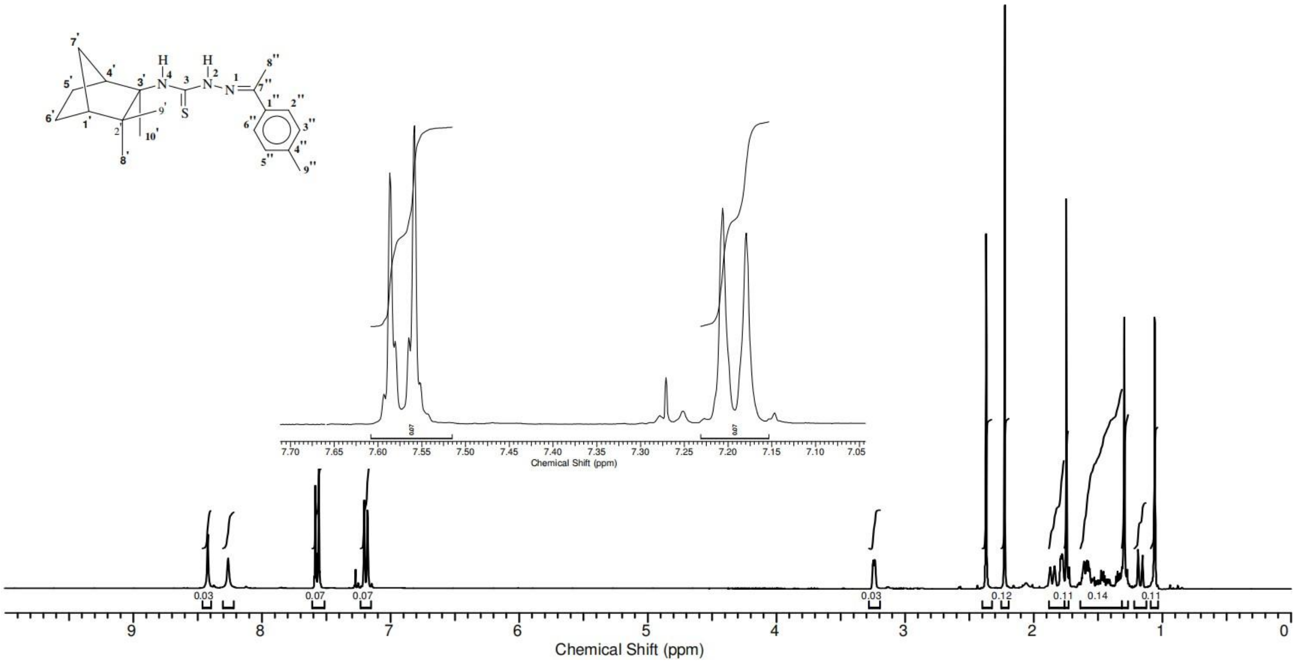

^{1}H NMR spectrum (300.06 MHz, $CDCl_3$) of compound **22**.

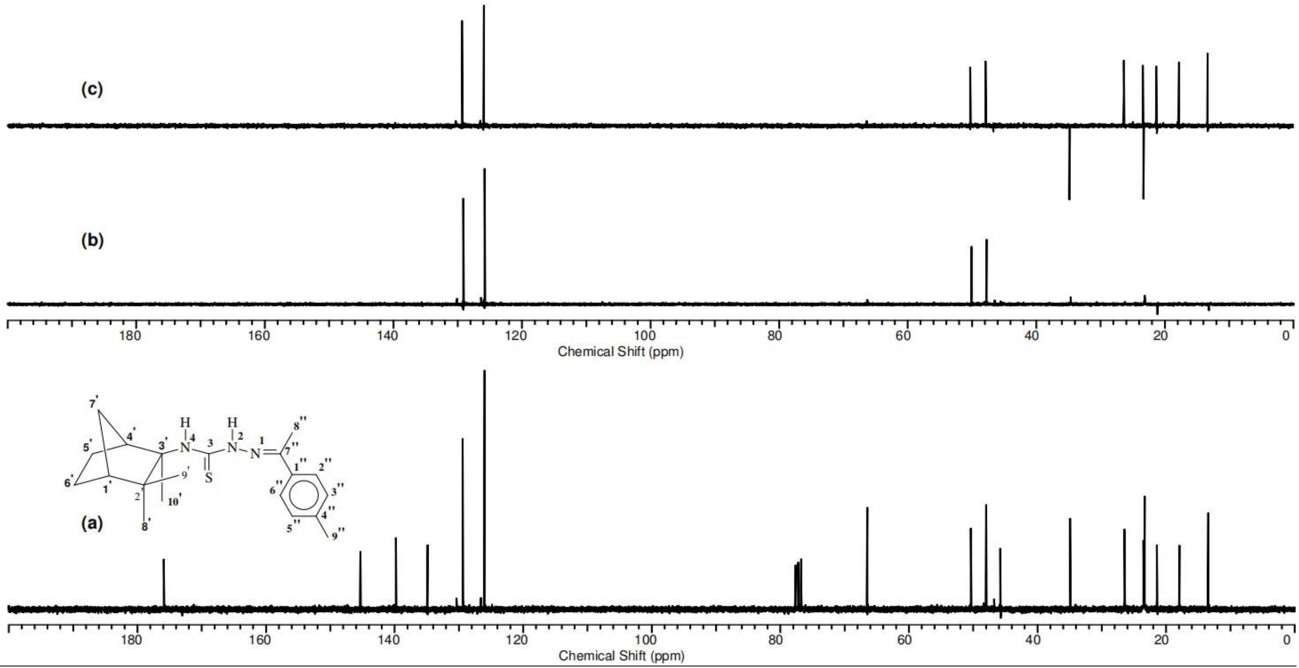

^{3}C(a)NMR/DEPT 90°(b) and DEPT 135°(c) spectra (75.45 MHz, $CDCl_3$) of compound **22**.

***p*-Fluoracetophenone (-)-camphene-based thiosemicarbazone (compound 23 in Fig 1)**

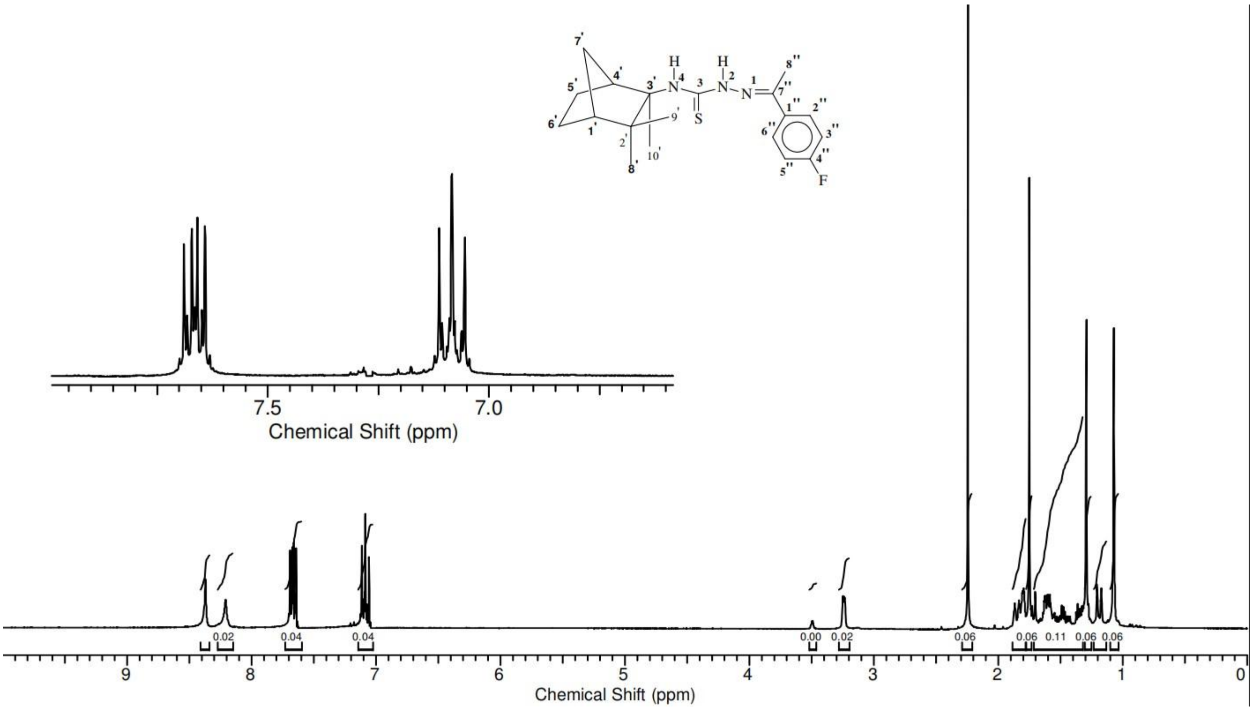

^{1}H NMR spectrum (300.06 MHz, $CDCl_3$) of compound **23**.

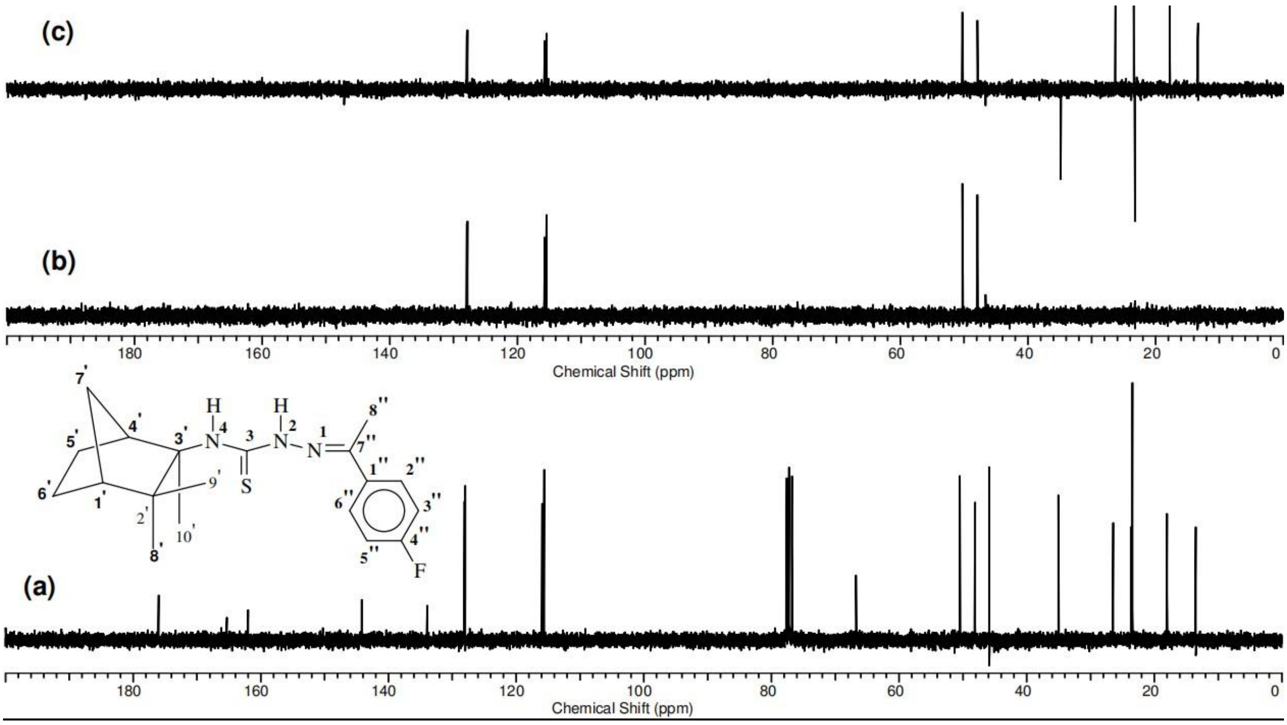

^{3}C(a)NMR/DEPT 90°(b) and DEPT 135°(c) spectra (75.45 MHz, $CDCl_3$) of compound **23**.

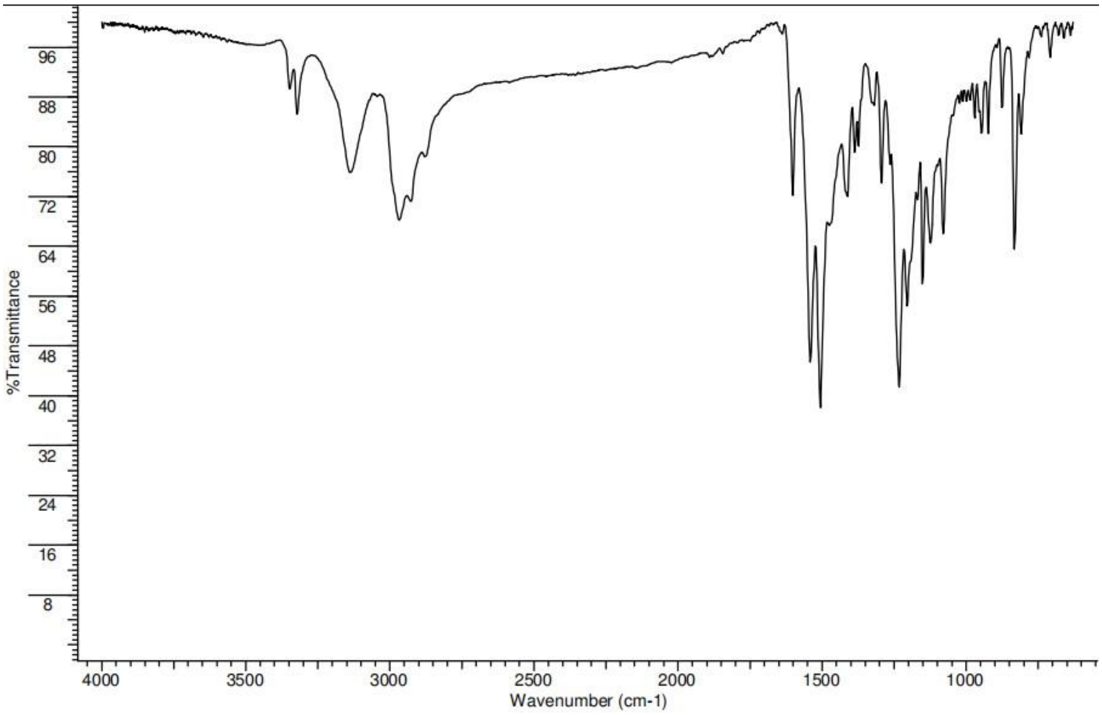

Infrared spectrum (KBr pellet) of compound **23**.

p-Hydroxyacetophenone (-)-camphene-based thiosemicarbazone (compound 24 in Fig 1)

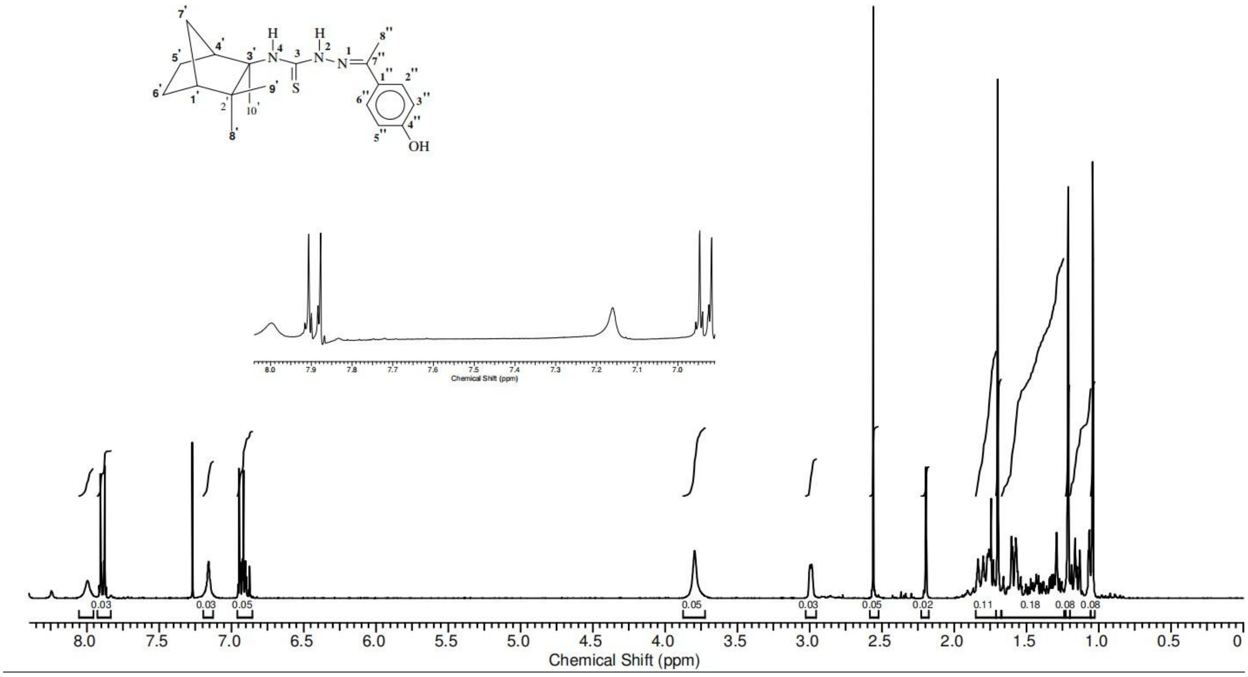

^{1}H NMR spectrum (300.06 MHz, $CDCl_3$) of compound **24**.

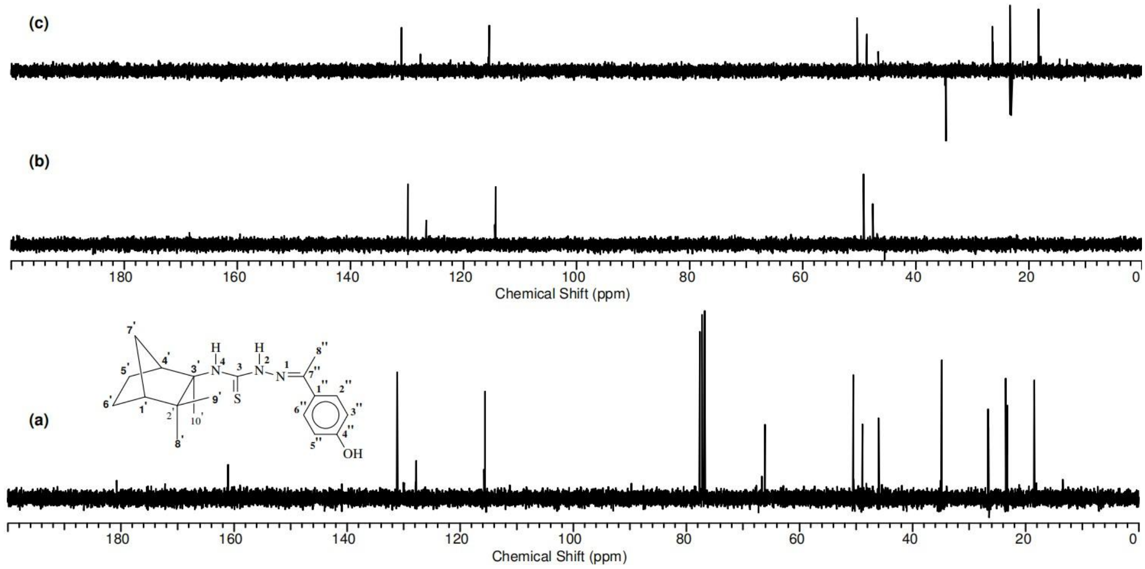

^{13}C(a)NMR/DEPT 90°(b) and DEPT 135°(c) spectra (75.45 MHz, $CDCl_3$) of compound **24**.

Thiophene-2-carboxyaldehyde (-)-camphene-based thiosemicarbazone (compound 25 in Fig 1)

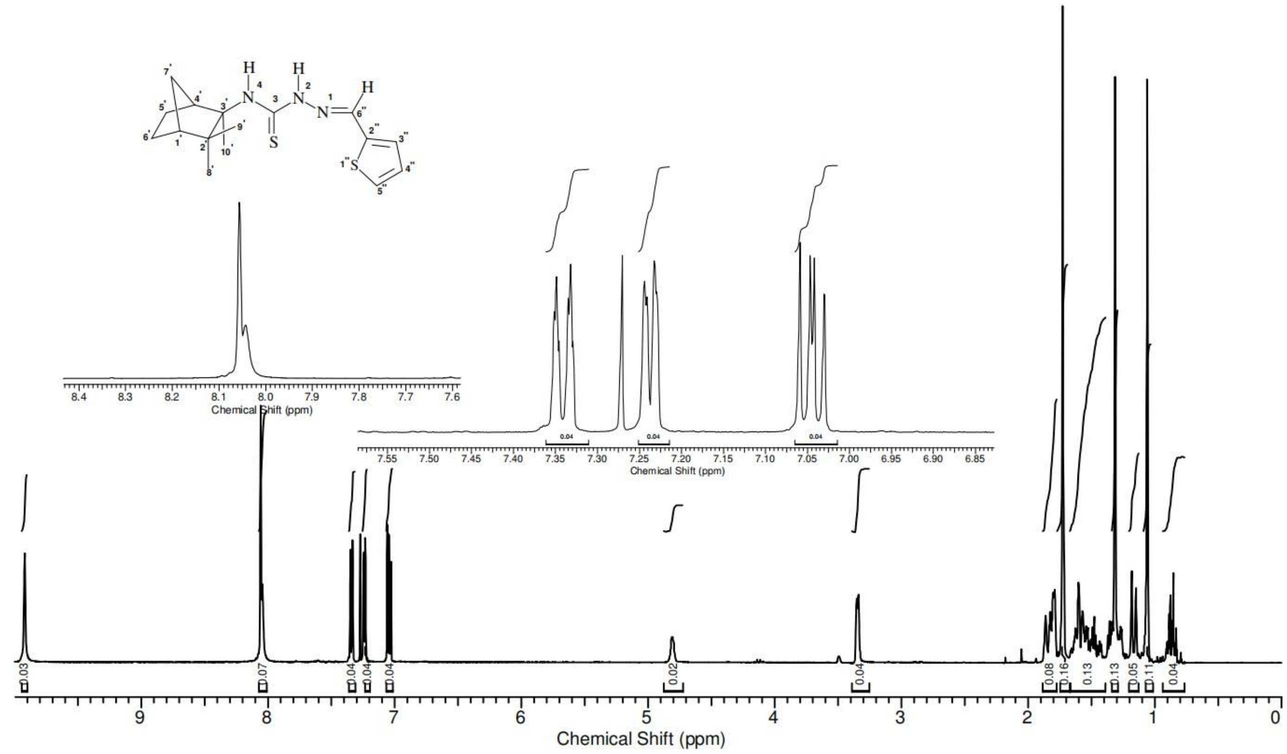

^{1}H NMR spectrum (300.06 MHz, $CDCl_3$) of compound **25**.

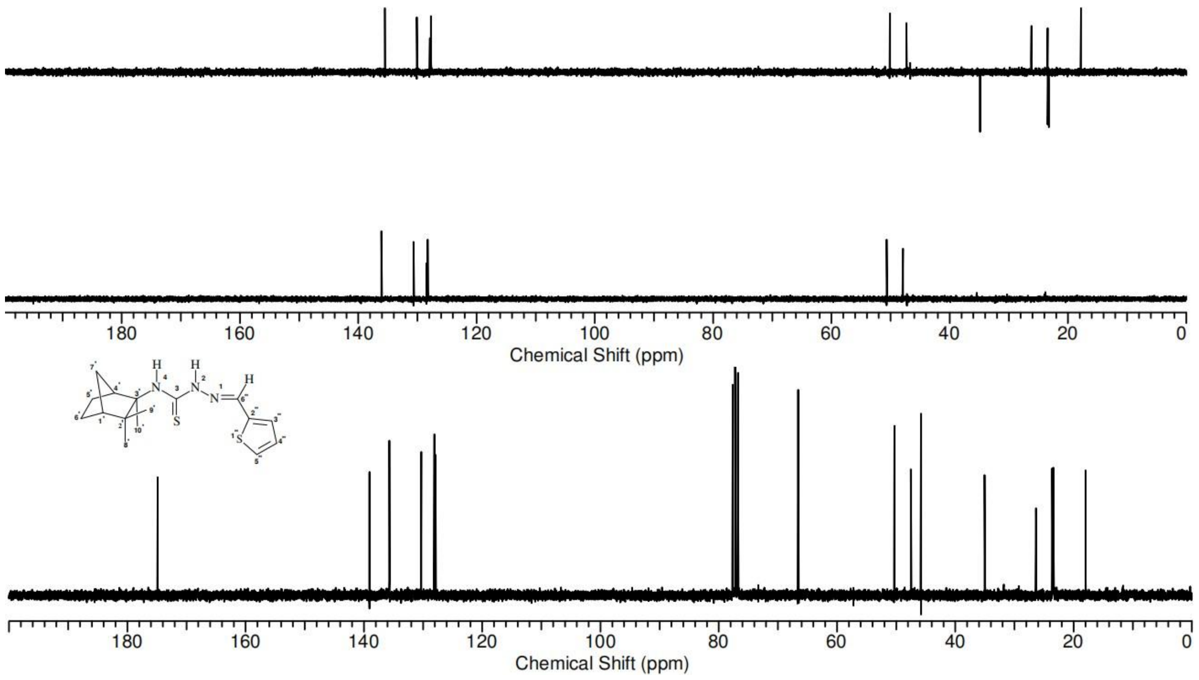

^{3}C(a)NMR/DEPT 90°(b) and DEPT 135°(c) spectra (75.45 MHz, $CDCl_3$) of compound **25**.

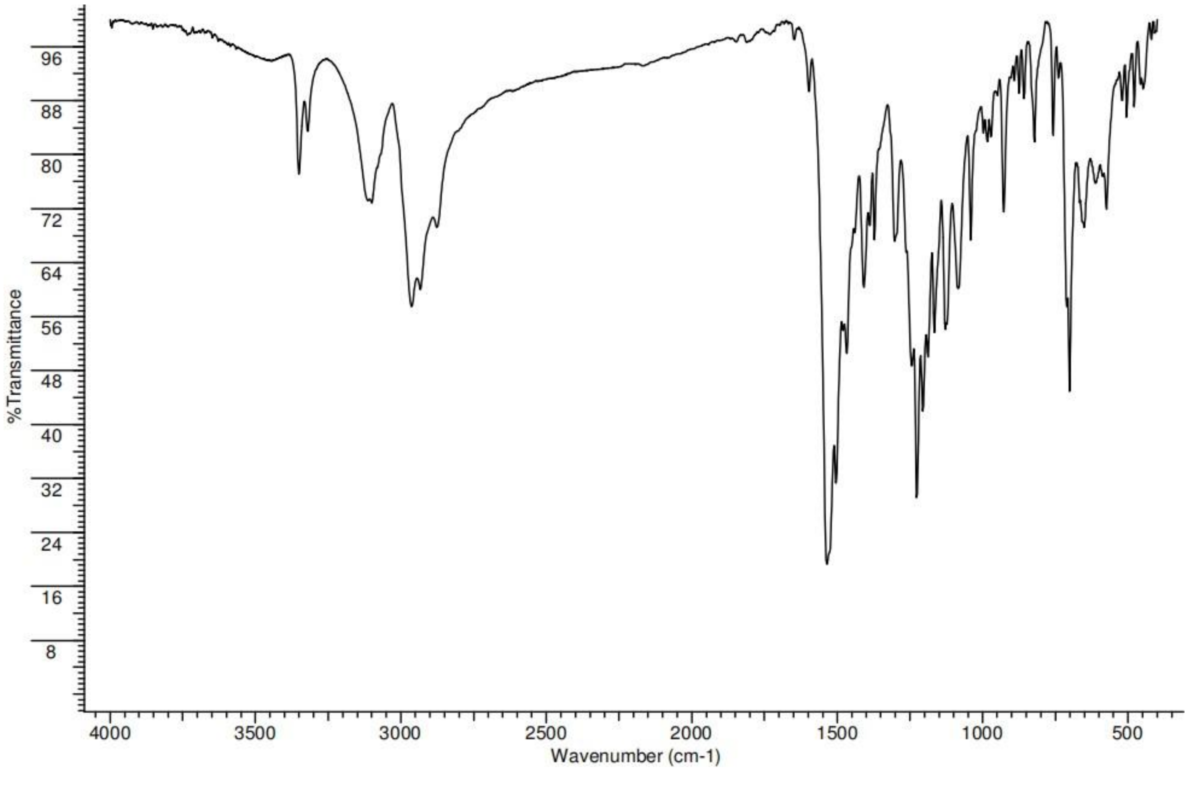

Infrared spectrum (KBr pellet) of compound **25**.

Furaldehyde (-)-camphene-based thiosemicarbazone (compound 26 in Fig 1)

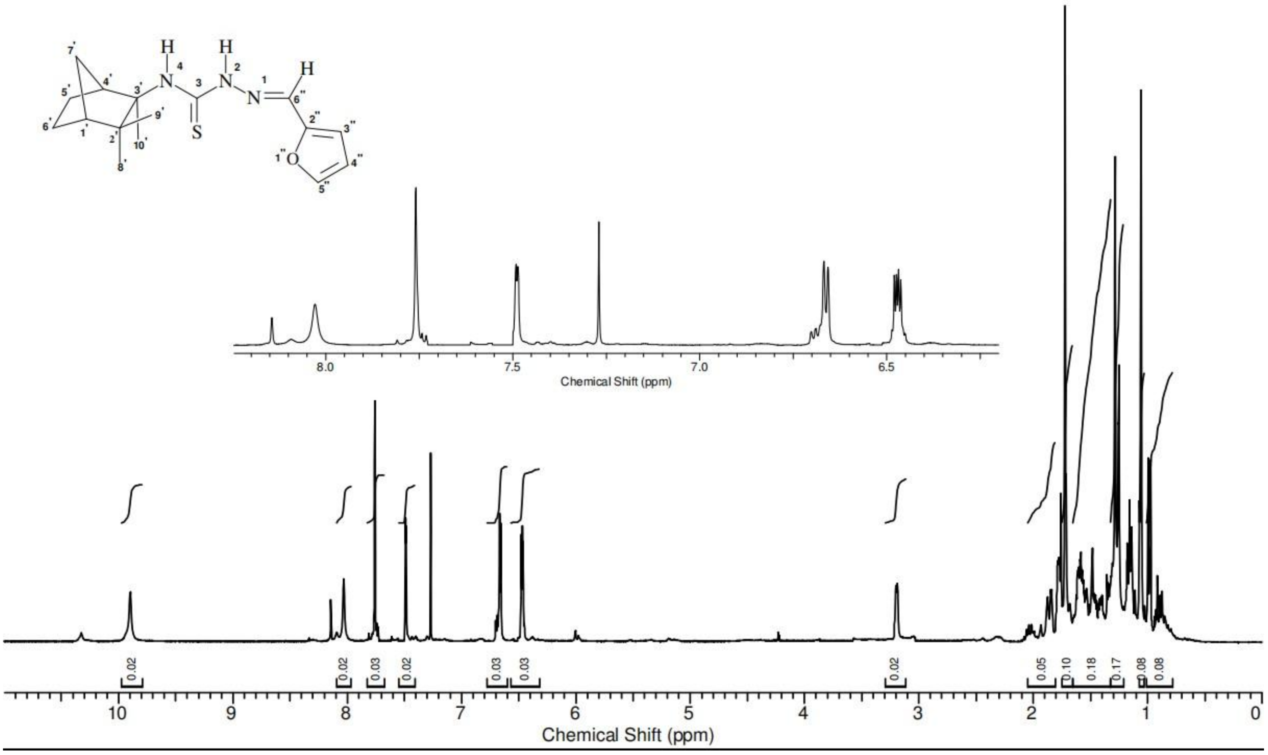

^{1}H NMR spectrum (300.06 MHz, $CDCl_3$) of compound **26**.

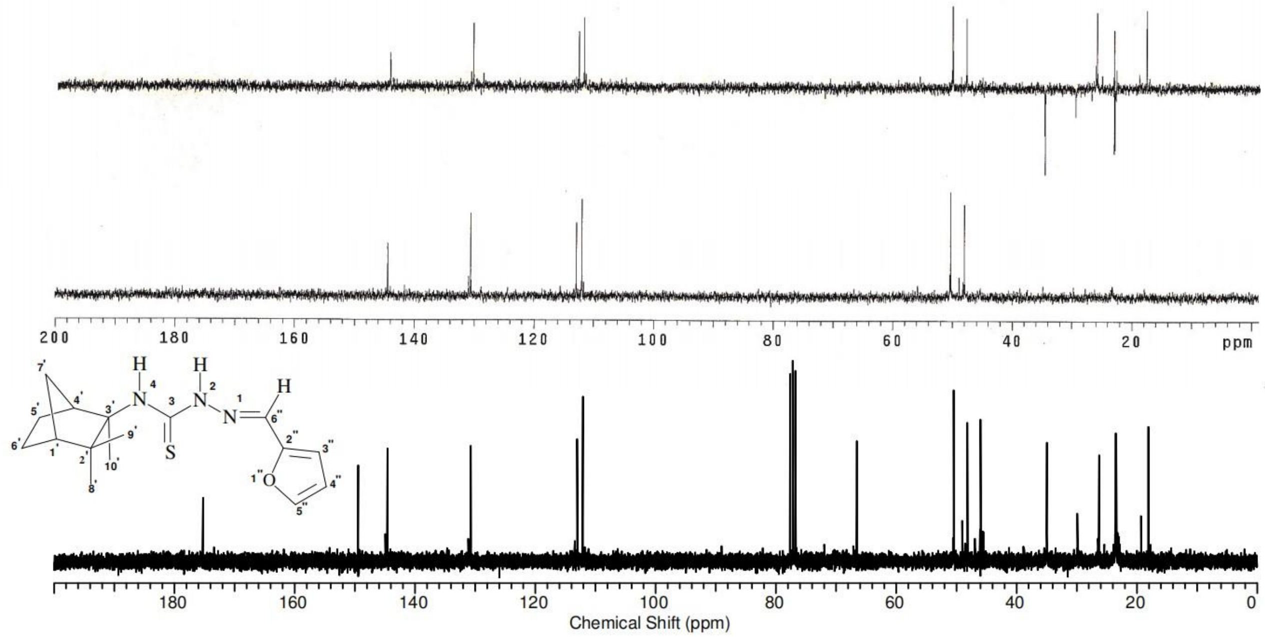

^{3}C(a)NMR/DEPT 90°(b) and DEPT 135°(c) spectra (75.45 MHz, $CDCl_3$) of compound **26**.

1H-pyrrole-2-carboxyaldehyde (-)-camphene-based thiosemicarbazone (compound 27 in Fig 1)

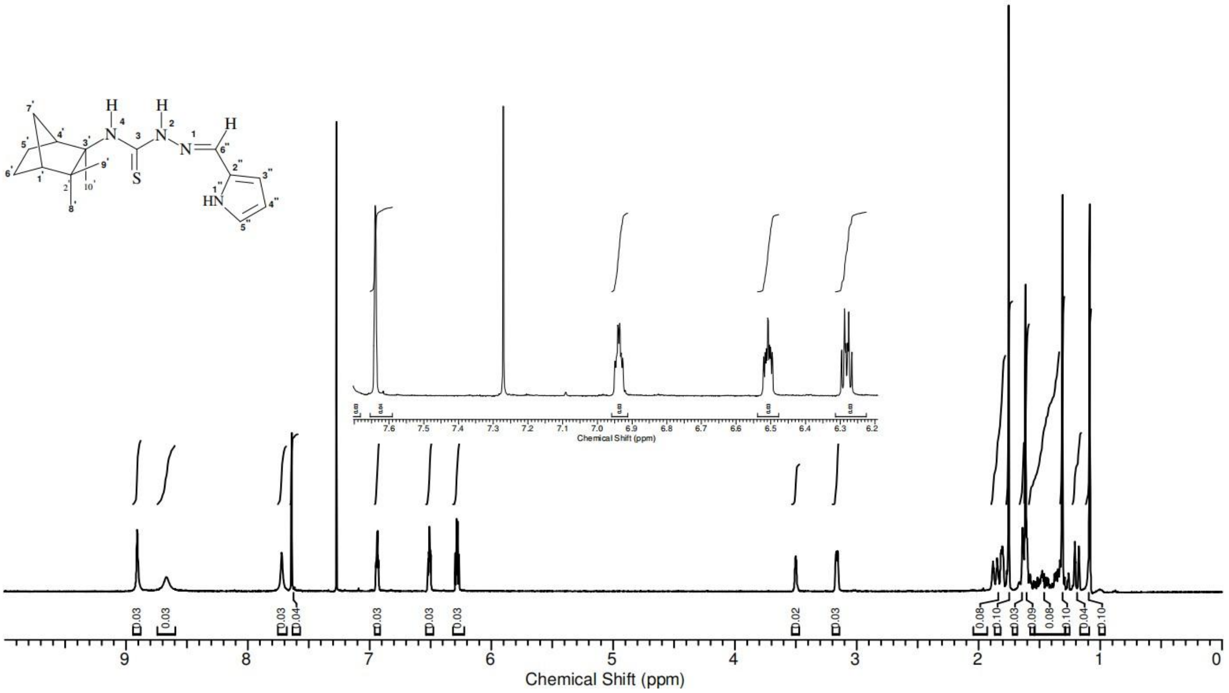

^{1}H NMR spectrum (300.06 MHz, $CDCl_3$) of compound **27**.

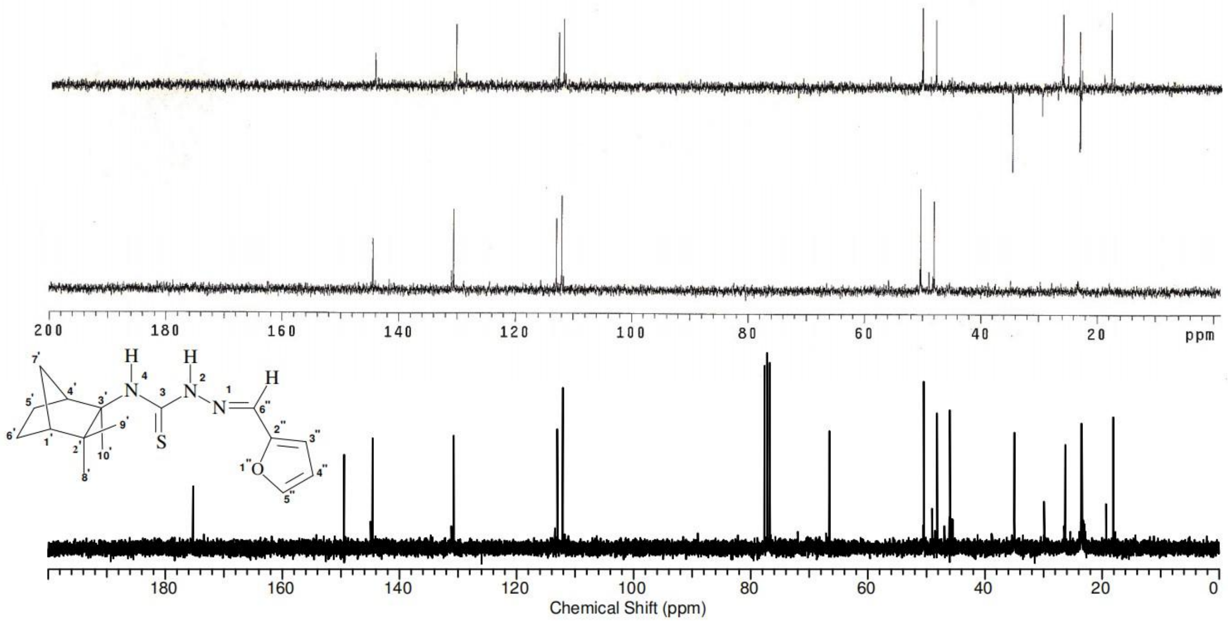

^{3}C(a)NMR/DEPT 90°(b) and DEPT 135°(c) spectra (75.45 MHz, $CDCl_3$) of compound **27**.

Benzophenone (-)-camphene-based thiosemicarbazone (compound 28 in Fig 1)

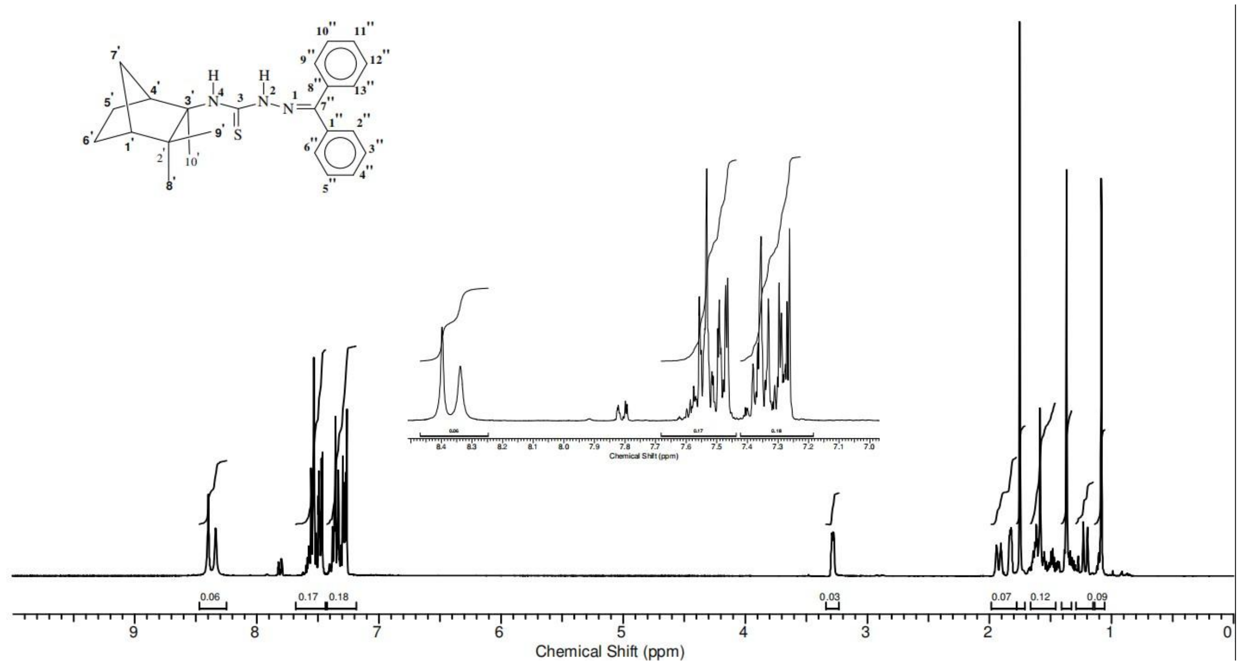

^{1}H NMR spectrum (300.06 MHz, $CDCl_3$) of compound **28**.

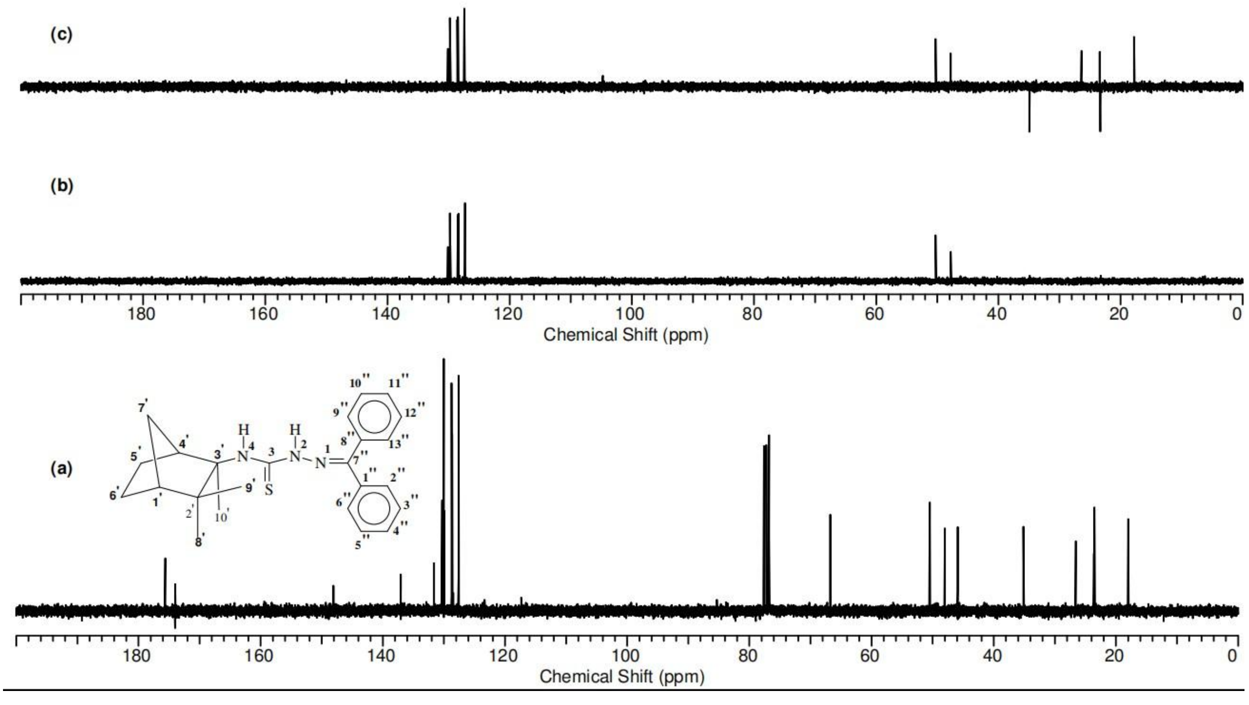

^{3}C(a)NMR/DEPT 90°(b) and DEPT 135°(c) spectra (75.45 MHz, $CDCl_3$) of compound **28**.

Cinnamaldehyde (-)-camphene-based thiosemicarbazone (compound 29 in Fig 1)

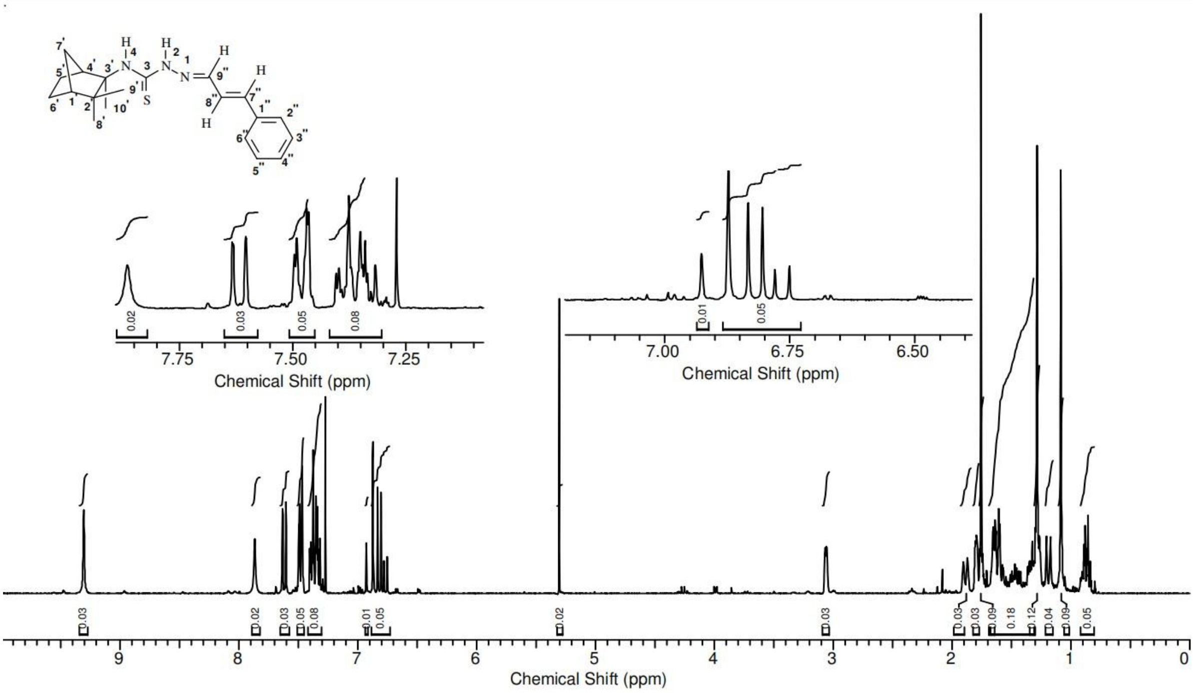

^{1}H NMR spectrum (300.06 MHz, $CDCl_3$) of compound **29**.

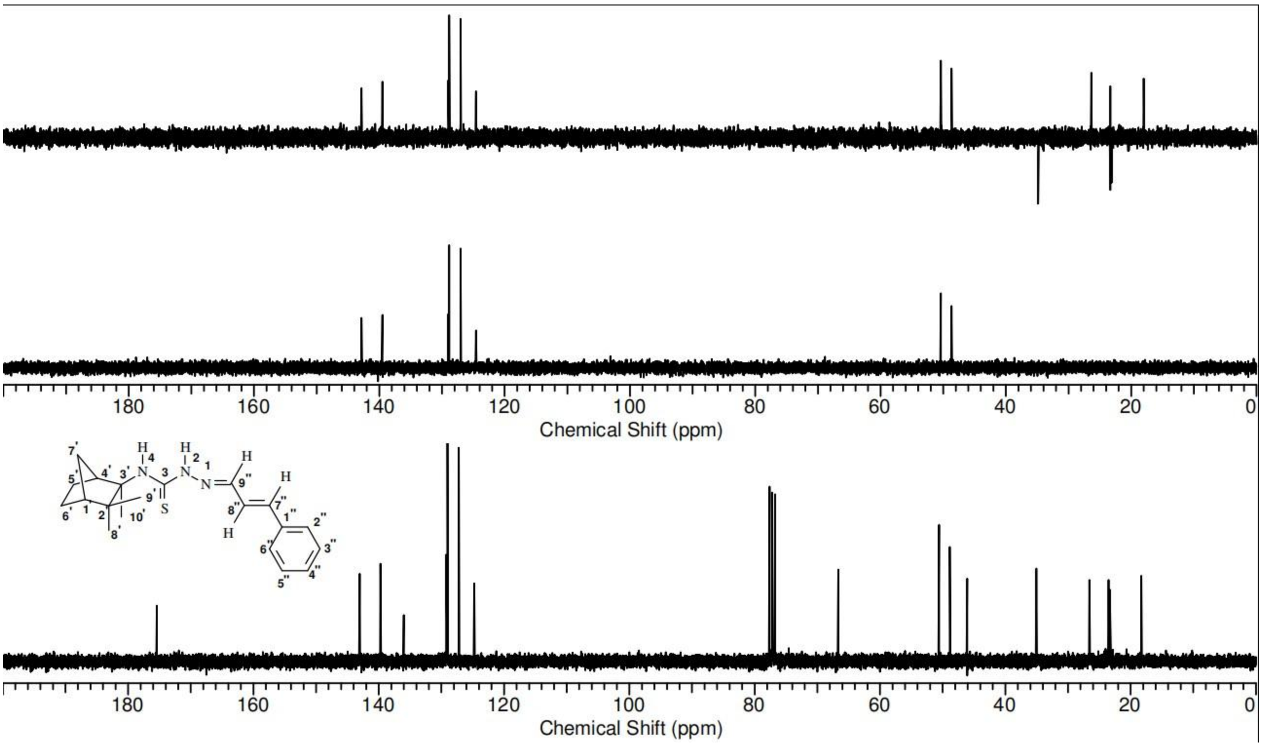

^{3}C(a)NMR/DEPT 90°(b) and DEPT 135°(c) spectra (75.45 MHz, $CDCl_3$) of compound **29**.

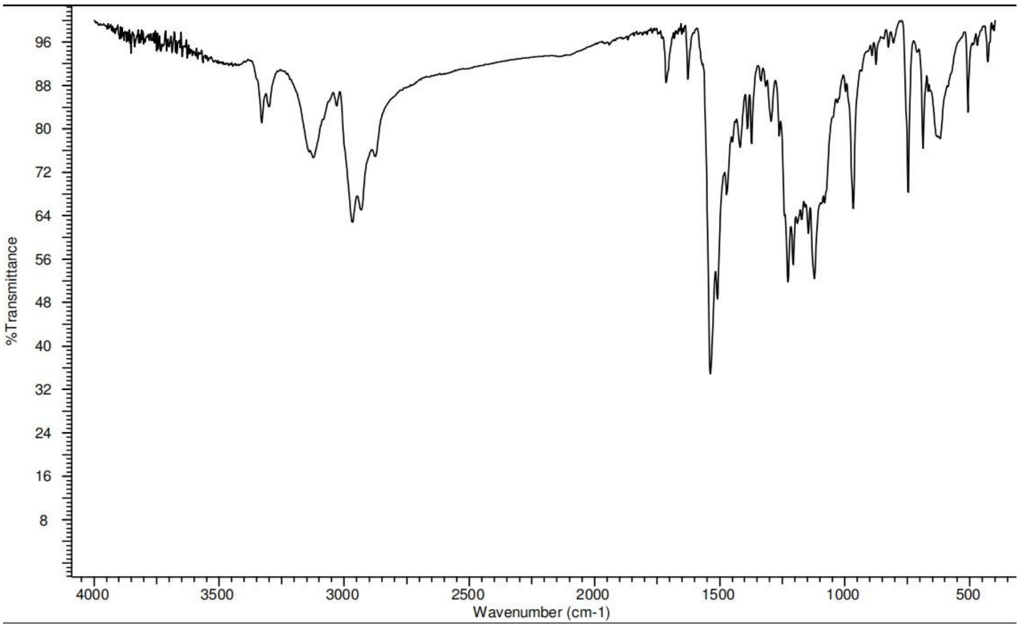

Infrared spectrum (KBr pellet) of compound **29**.

Menthone (-)-camphene-based thiosemicarbazone (compound 30 in Fig 1)

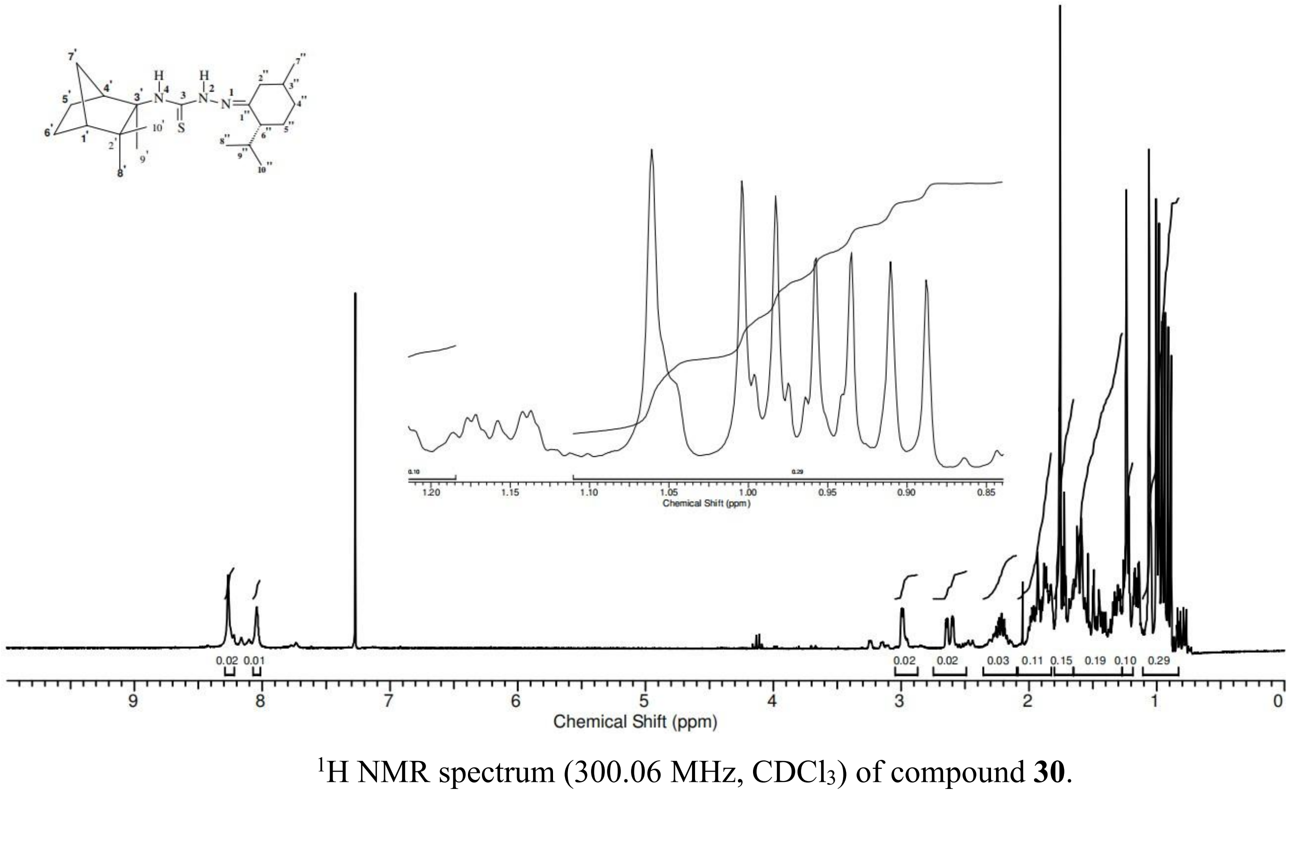

^{1}H NMR spectrum (300.06 MHz, $CDCl_3$) of compound **30**.

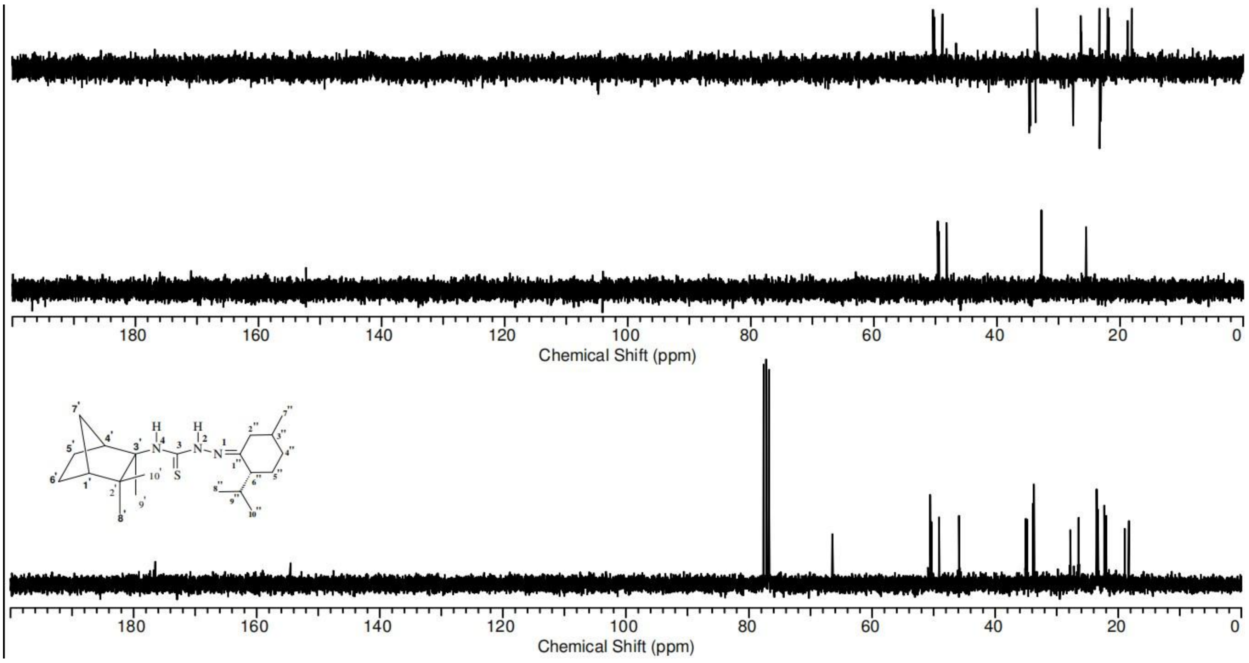

^{3}C(a)NMR/DEPT 90°(b) and DEPT 135°(c) spectra (75.45 MHz, $CDCl_3$) of compound **30**.

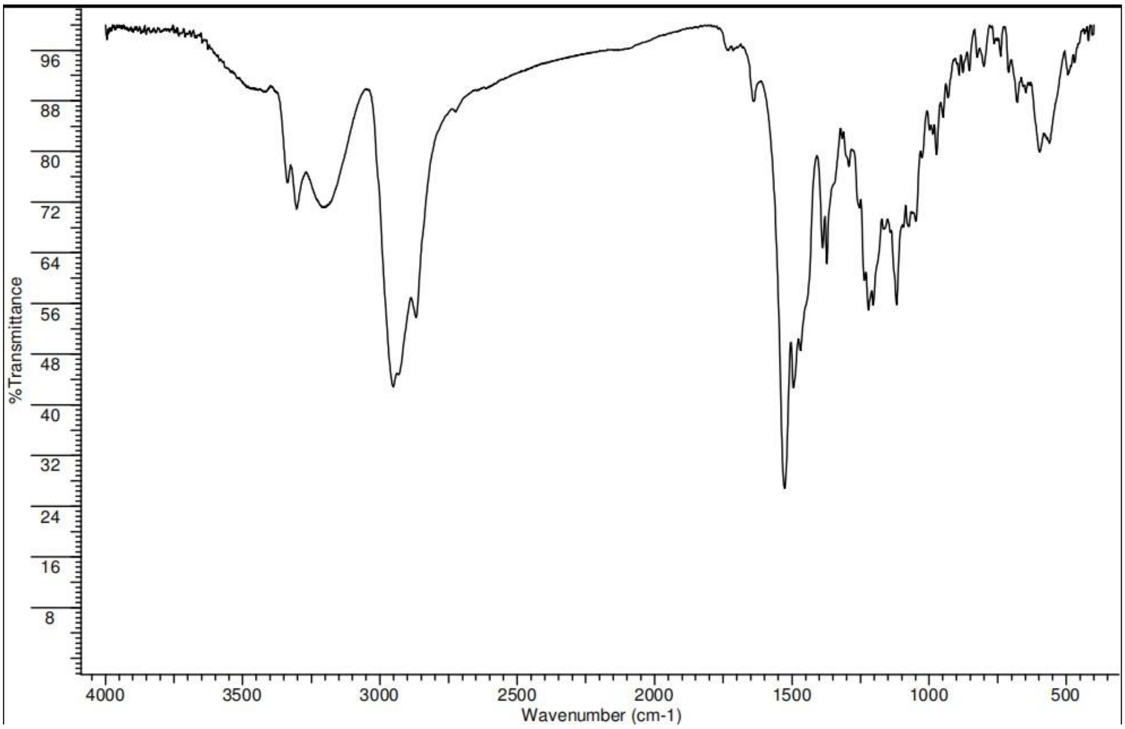

Infrared spectrum (KBr pellet) of compound **30**.

Ethylpyruvate (-)-camphene-based thiosemicarbazone (compound 31 in Fig 1)

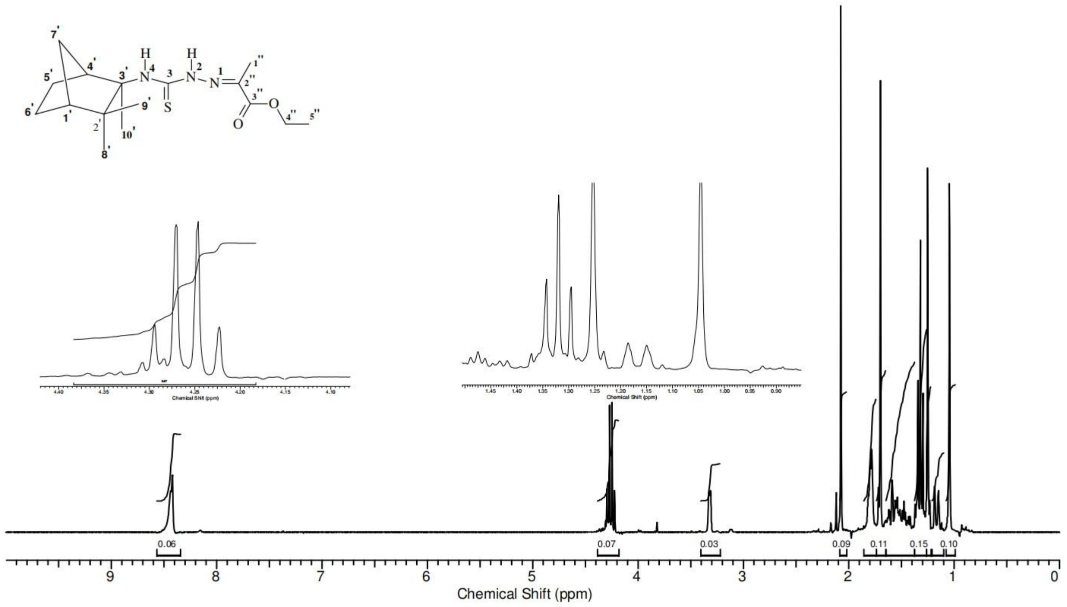

^{1}H NMR spectrum (300.06 MHz, $CDCl_3$) of compound **31**.

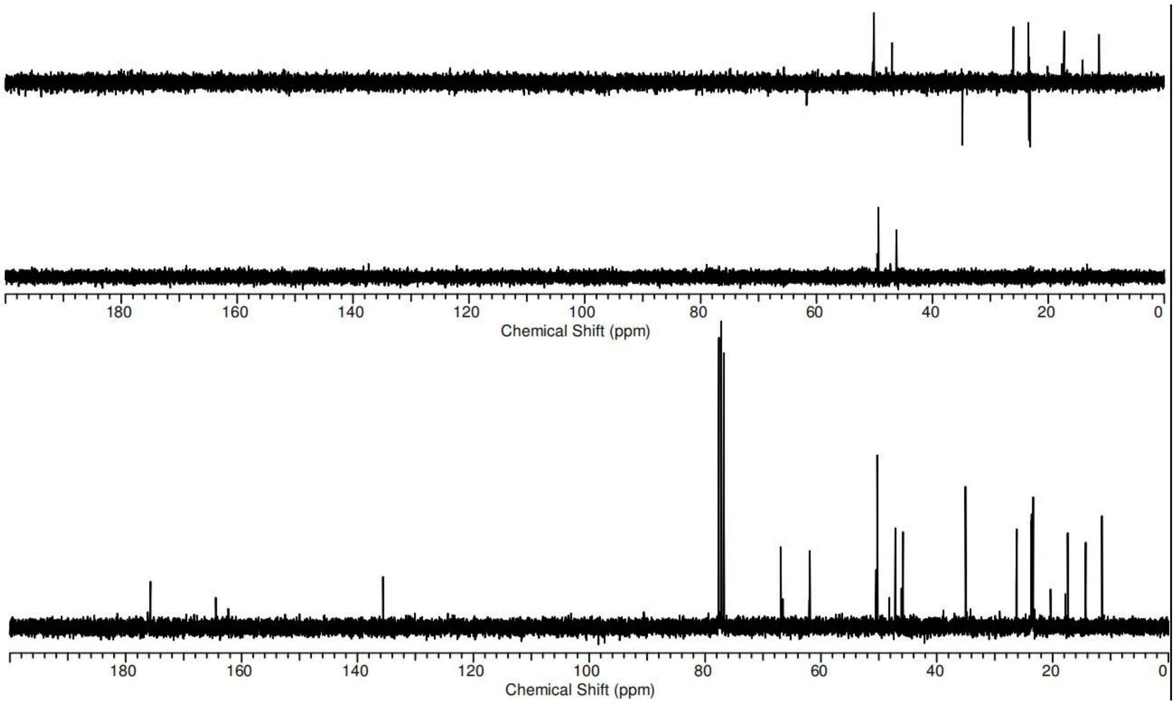

^{3}C(a)NMR/DEPT 90°(b) and DEPT 135°(c) spectra (75.45 MHz, $CDCl_3$) of compound **31**.

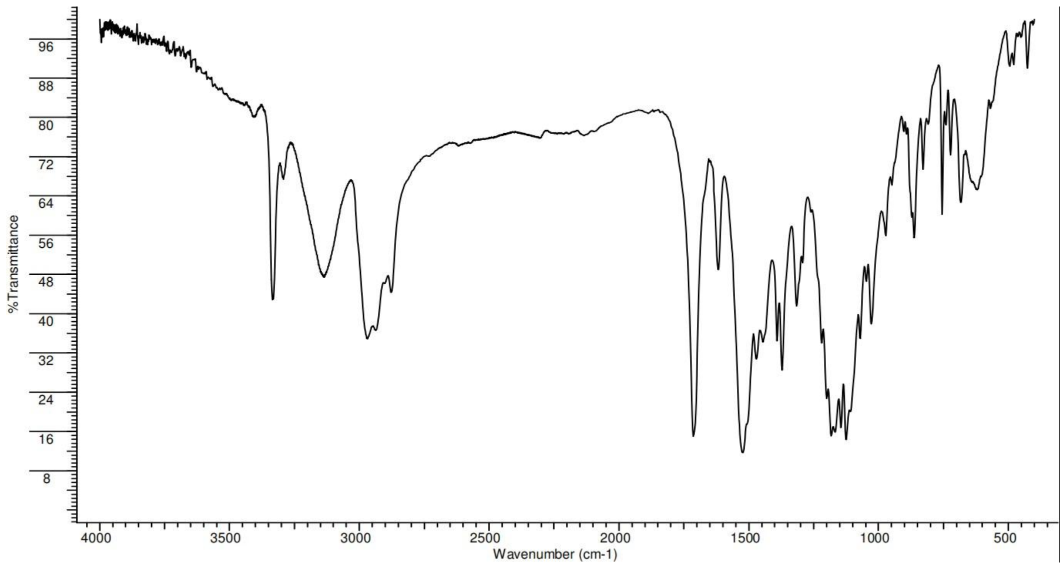

Infrared spectrum (KBr pellet) of compound **31**.
