# Supplementary material for: In vitro antiproliferative and apoptotic effects of thiosemicarbazones based on (-)-camphene and R-(+)-limonene in human melanoma cells
Source: PLoS One. 2023 Nov 30;18(11):e0295012. doi: 10.1371/journal.pone.0295012 (PMC10688736; doi:10.1371/journal.pone.0295012)
Supplement: S2 File — Optimization of kinetic conditions for the enzymatic reaction of caspases. (PDF) [file pone.0295012.s002.pdf]

S2 Standardization of conditions for the colorimetric enzymatic assay of caspases 2, 3, 6, 8 and 9.

To accomplish this, 50 µL duplicates of the supernatant were combined with 50 µL of reaction buffer containing 10 mM DTT. The resulting mixture was incubated in the dark at 37°C for 2, 15, and 30 hours with 200 µM peptide substrates. The substrates used were VDVAD (Val-Asp-Val-Ala-Asp- for caspase-2), DEVD (Asp-Glu-Val-Asp for caspase-3), VEID (Val-Glu-Ile-Asp for caspase-6), IETD (Ile-Glu-Thr-Asp- for caspase-8), and LEHD (Leu-Glu-His-Asp- for caspase-9), and were labeled at their C-terminal region with the chromophore *p*-nitroaniline (*p*NA). Subsequently, 100 µL of each sample were transferred in duplicate to the wells of a microtiter plate. Absorbance was measured at 405 nm using a microplate reader (Biotek, USA). The experiments were performed on two separate occasions. Additionally, a standard curve was plotted to calculate the concentration of the chromophore *p*-nitroaniline released by caspase activity. A 10 mM stock solution of *p*-nitroaniline in DMSO was prepared, and solutions containing 200 µM, 100 µM, 50 µM, 20 µM, 10 µM and 5 µM were made in the lysis buffer provided by the kit. The results were plotted using GraphPad Prism 4.00 for Windows (GraphPad Software, San Diego,USA, www.graphpad.com).

The following graphs display the kinetics performed with the caspases and the standard curve obtained for calculating the concentration of *p*-nitroaniline released by the enzymatic reaction.

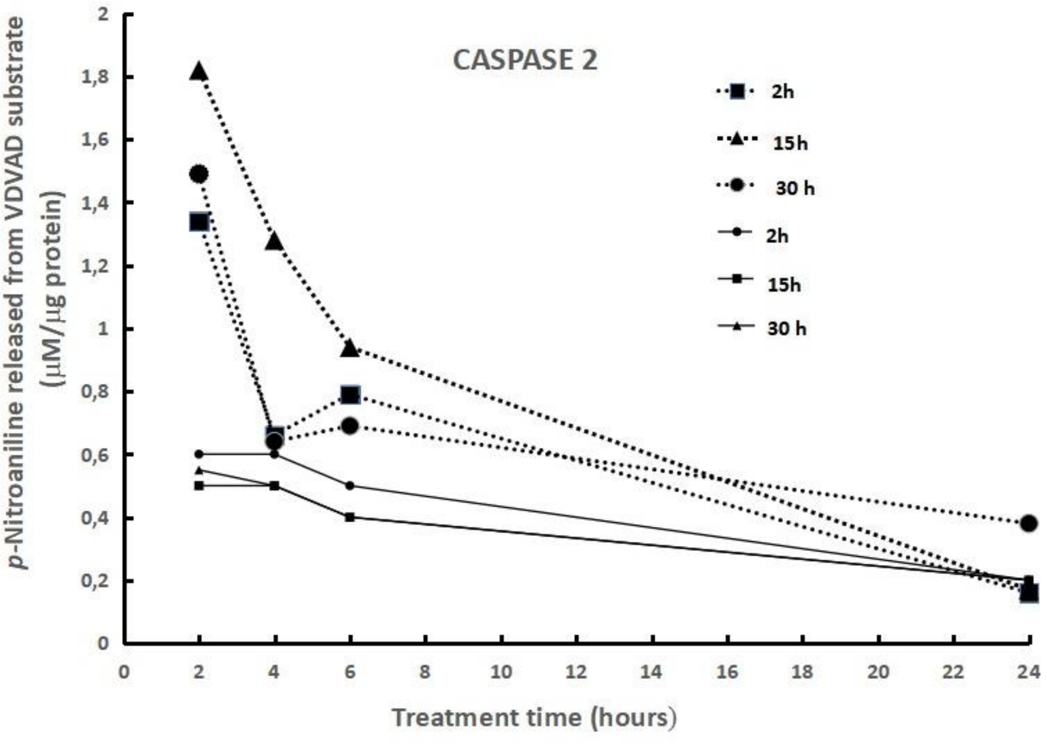

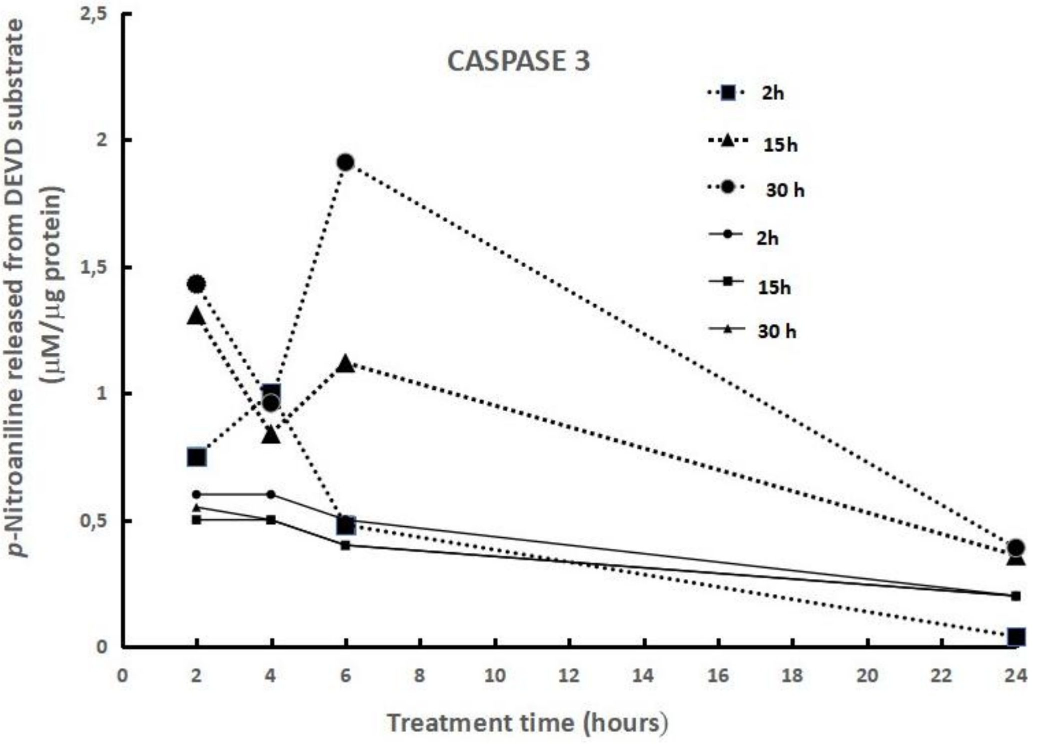
CASPASE 3
2h
15h
30 h
2h
15h
30 h
p-Nitroaniline released from DEVD substrate (µM/µg protein)
Treatment time (hours)
0
0,5
1
1,5
2
2,5
0
2
4
6
8
10
12
14
16
18
20
22
24

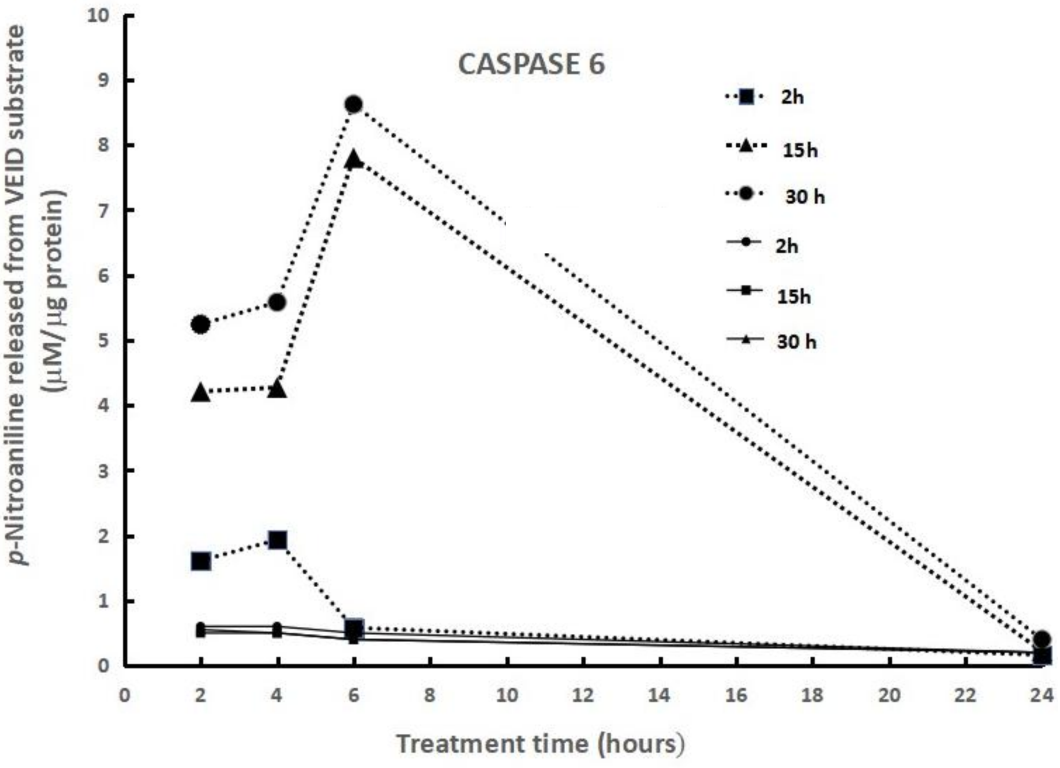
CASPASE 6
2h
15h
30 h
2h
15h
30 h
p-Nitroaniline released from VEID substrate (µM/µg protein)
Treatment time (hours)
0
1
2
3
4
5
6
7
8
9
10
0
2
4
6
8
10
12
14
16
18
20
22
24

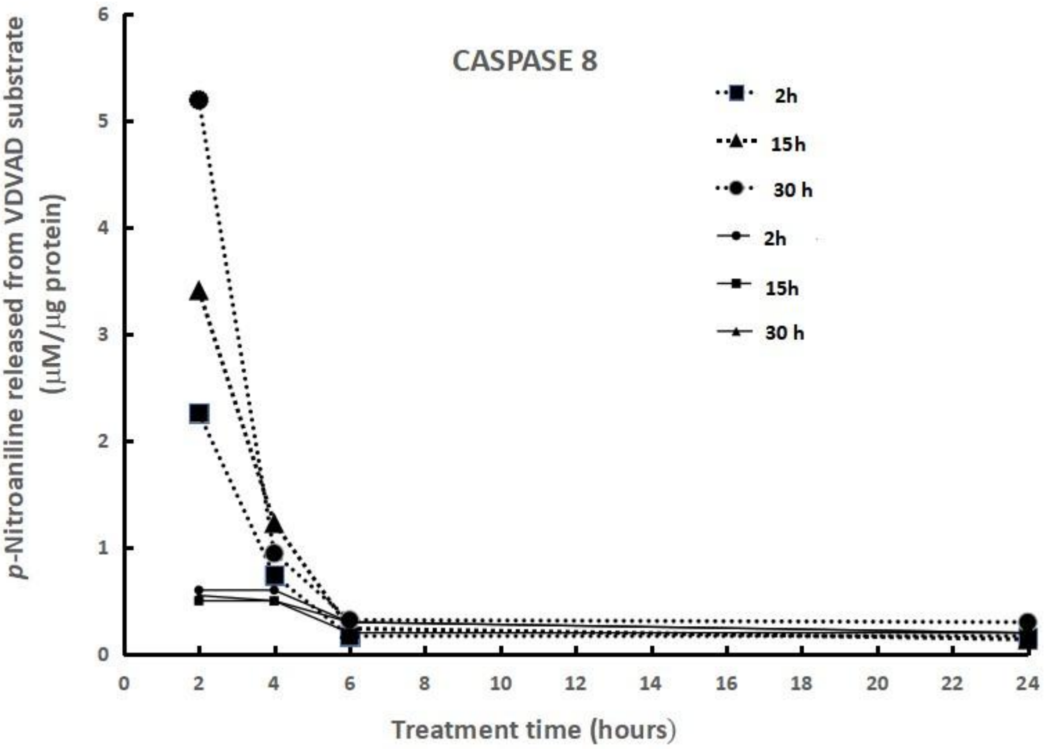

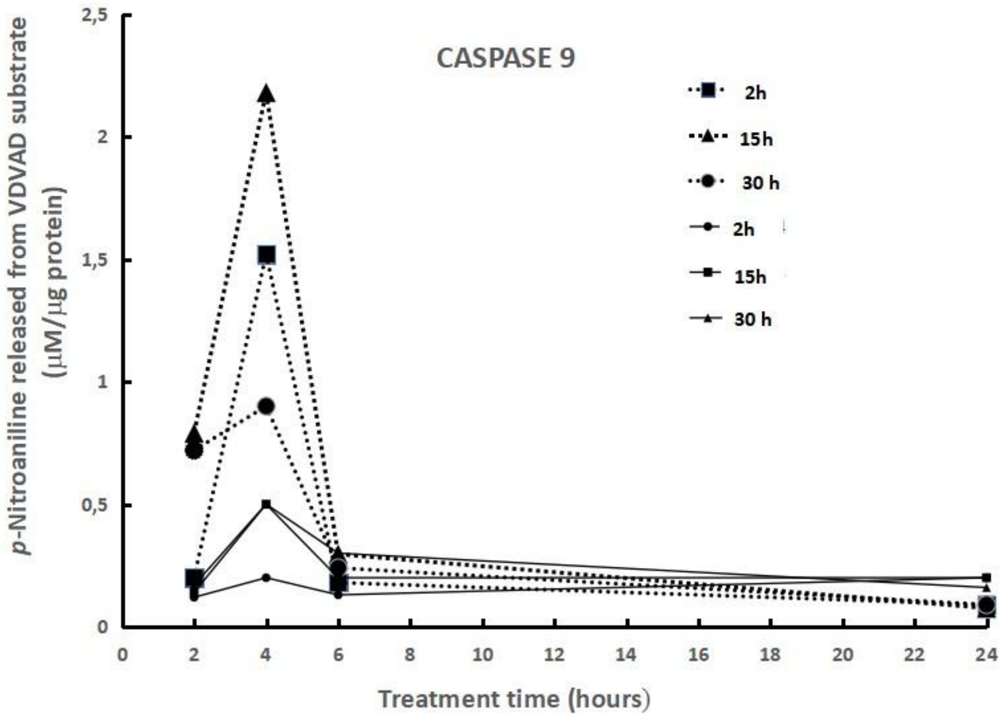

Kinetics of caspase 2, 3, 6, 8, and 9 activity. Exponentially growing SK-MEL-37 cells were treated with a 100 µM concentration of benzaldehyde (-)-camphene-based thiosemicarbazone for 2, 4, 6, and 24 hours. Optimization of caspase 2, 3, 6, 8, and 9 reactions was achieved by incubating 200 µM peptide substrates for 2 (■), 15 (▲), 30 (●) hours at 37ºC, to determine the optimal incubation time for the treated (dashed line) or control (solid line) samples.

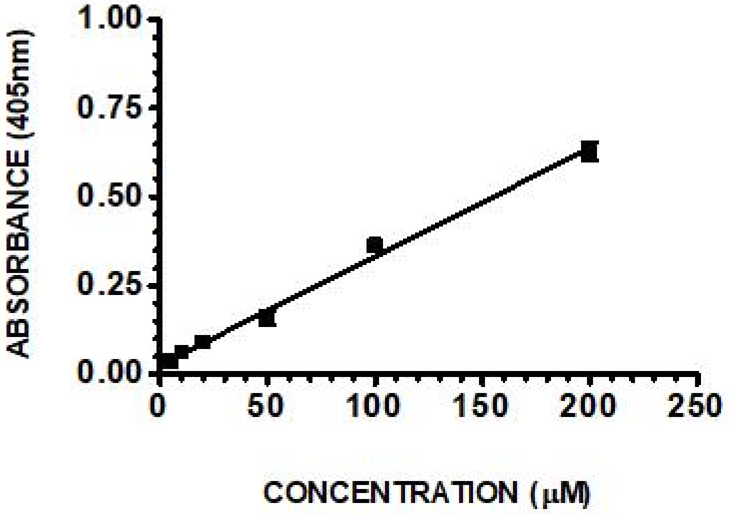

Standard curve for calculating the concentration of *p*-nitroaniline. The curve was generated using a 10 mM stock solution of *p*-nitroaniline in DMSO, and solutions containing 200 µM, 100 µM, 50 µM, 20 µM, 10 µM and 5 µM were made in the lysis buffer provided by the kit. Absorbance was measured at 405 nm using a microplate reader (Biotek, USA), and the results were plotted to create the standard curve. The data presented here are representative of one of the two independent experiments conducted.
