# Supplementary material for: In vitro antiproliferative and apoptotic effects of thiosemicarbazones based on (-)-camphene and R-(+)-limonene in human melanoma cells
Source: PLoS One. 2023 Nov 30;18(11):e0295012. doi: 10.1371/journal.pone.0295012 (PMC10688736; doi:10.1371/journal.pone.0295012)
Supplement: S1 Raw image — (PDF) [file pone.0295012.s003.pdf]

S3 Raw eletroforese gel image

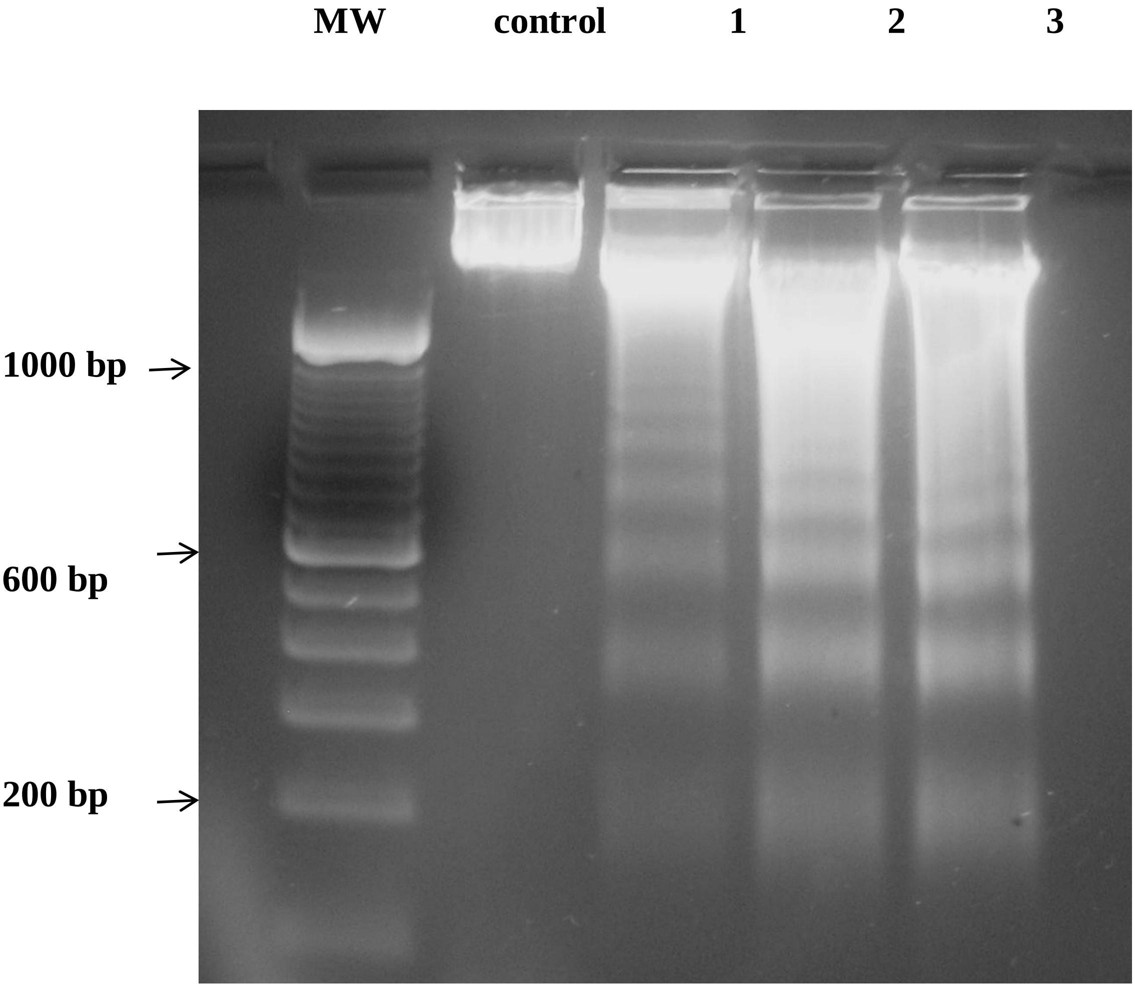

Analysis of DNA fragmentation by agarose gel electrophoresis. SK-MEL-37 cells were treated with the indicated compounds at a concentration of 100 μM. After 24 hours of incubation, both attached and floating cells were collected, and DNA was extracted and analyzed by 2% agarose gel electrophoresis containing 0.5% thidium bromide. The fluorescent image was obtained using a transilluminator UV/white light and captured with a Canon PowerShot A85 camera. The image shows genomic DNA fragmentation in multiples of 200 bp. 1: benzaldehyde (-)-camphene-based thiosemicarbazone; 2: *m*-chlorobenzaldehyde (-)-camphene-based thiosemicarbazone; 3: *m*-nitrobenzaldehyde (-)-camphene-based thiosemicarbazone. Molecular weight marker (MW) 100 bp (Invitrogen).
