# Supplementary material for: In vitro antiproliferative and apoptotic effects of thiosemicarbazones based on (-)-camphene and R-(+)-limonene in human melanoma cells
Source: PLoS One. 2023 Nov 30;18(11):e0295012. doi: 10.1371/journal.pone.0295012 (PMC10688736; doi:10.1371/journal.pone.0295012)
Supplement: Attachment — Submitted filename: Response to Reviewers.docx [file pone.0295012.s004.docx]

Lidia Andreu Guillo

[guillo@ufg.br](mailto:guillo@ufg.br)

PONE-D-23-14168

*In vitro* antiproliferative and apoptotic effects of thiosemicarbazones based on (-)-camphene and R-(+)-limonene in human melanoma cells

Dear Dr. Wagdy Mohamed Eldehna and Reviewers,

We would like to express our sincere gratitude for the thorough review of our manuscript titled "*In vitro* antiproliferative and apoptotic effects of thiosemicarbazones based on (-)-camphene and R-(+)-limonene in human melanoma cells".

We commence this rebuttal letter by addressing the points raised in the “Journal Requirements”, and subsequently in the “Reviewers' comments”.

1. “Please ensure that your manuscript meets PLOS ONE's style requirements, including those for file naming.”

**Response**: We have meticulously reviewed our manuscript to align with PLOS ONE's style requirements, encompassing both the formatting of the manuscript body and adherence to the stipulated file naming guidelines outlined in the provided template.

2. “Thank you for stating in your Funding Statement:...”

**Response**: We appreciate the opportunity to clarify the funding support for our study. Although the support we received was in the form of master's and doctoral research fellowships from CNPq, we understand that these fellowships qualify as a type of funding for the research. We apologize for any confusion caused by indicating "NO" under "funded studies" during the submission process. The Funding Statement has been revised to accurately reflect that the study received support from CNPq through research fellowships for the first two authors, PROS and DCSP. In addition, we would like to clarify the nature of the funding support received for our study. The support provided by CNPq was in the form of research fellowships, which supported the academic pursuits of the first two authors, PROS and DCSP, during their master's and doctoral studies. We acknowledge that while the funding assisted in covering personal expenses, it did not entail direct salaries. Importantly, the funding did not influence or contribute to the study design, data collection and analysis, decision to publish, or preparation of the manuscript. The roles of these authors in the research are detailed in the 'author contributions' section of the manuscript.

Therefore, the following Funding Statement was included in the Cover Letter to allow for modifications by the system:

**Funding**: This study was made possible through the research fellowships awarded to PROS and DCSP by the National Council for Scientific and Technological Development (CNPq) of Brazil. These fellowships supported PROS during her master's studies and DCSP during her doctoral studies, covering personal expenses during the research period, and they did not include direct salaries. CNPq played no additional role in the study design, data collection and analysis, decision to publish, or manuscript preparation. No additional external finding was received for this study.

3. “We note that you have stated that you will provide repository information for your data at acceptance. Should your manuscript be accepted for publication, we will hold it until you provide the relevant accession numbers or DOIs necessary to access your data. If you wish to make changes to your Data Availability statement, please describe these changes in your cover letter and we will update your Data Availability statement to reflect the information you provide.”

**Response**: We appreciate the attention to the Data Availability statement for our manuscript. Upon revisiting our initial submission, we realized that we had selected: “All relevant data are within the manuscript and its Supporting Information files”. We would like to confirm that all pertinent data for our study have been included directly within the manuscript and its supporting information files. Hence, there are no separate accession numbers or repository information to provide. We are thankful for the opportunity to clarify this aspect and ensure that the data accessibility is appropriately communicated.

4. “PLOS ONE now requires that authors provide the original uncropped and unadjusted images underlying all blot or gel results...”

“In your cover letter, please note whether your blot/gel image data are in Supporting Information or posted at a public data repository, provide the repository URL if relevant, and provide specific details as to which raw blot/gel images, if any, are not available. Email us at plosone@plos.org if you have any questions.”

**Response**: We have included the original, uncropped, and unadjusted gel image in our Supporting Information file, labeled as a PDF file named S3_File_raw_gel image. Furthermore, in our cover letter, we have explicitly noted and clarified the location of our original gel image data. Regarding the requirements for blot/gel image data, in our revised submission, we have ensured that all figures adhere to the journal's guidelines for blot/gel reporting and figure preparation. No raw blot/gel images are missing, and we have provided the necessary details in the Supporting Information file. We would like to confirm that our gel image data are included in the Supporting Information, and no data are posted in a public data repository.

We will now address Reviewers’ comments:

**Points raised by Reviewer #1.**

1. “The language of the manuscript should be extensively revised.”

Response: We appreciate your feedback. The manuscript has undergone a comprehensive review, with special attention to language refinement to meet the highest readability and comprehension standards. Furthermore, we've taken the additional step of submitting the manuscript to AJE (American Journal Experts) for professional editing and formatting, which we believe will further enhance its clarity and overall quality.

2. “ Some symbols and words should be italic form as heteroatoms of the chemical names and in vitro.”

**Response**: We have carefully reviewed the manuscript and addressed the issue regarding italics for symbols and words, particularly for heteroatoms in chemical names and 'in vitro'. In our revised manuscript, we have ensured that these elements are properly italicized to improve clarity and conformity with the journal's style guidelines.Additionally, the editing and formatting services provided by AJE have contributed to this improvement.

3. “Abstract should be more concise.”

**Response**:. We appreciate your feedback on the abstract. We have carefully reviewed and revised the abstract to make it more concise and have successfully reduced its length from 349 words to 244 words. We believe this revision enhances the clarity and conciseness of the abstract while still effectively summarizing the key findings of our study.

4. “Some words start with capital letters although being in the middle of the sentence. Please check all manuscript.”

**Response**: We have carefully reviewed the entire manuscript to identify instances where words in the middle of sentences start with capital letters inappropriately. As an example, we noticed that compounds names in the middle of the sentence: “Among these, Benzaldehyde (-)-camphene-based, p-Methoxy, o-Chloro, p-Chloro, p-Nitro, p-Dimethylamine, and p-Hydroxy...” were written with initial capital letters, which is not in line with proper style. Therefore, we have made the necessary adjustments to ensure that all such words are in lowercase within sentences throughout the manuscript.

5. “The introduction section is not comprehensive. It should comprise the background, aim of the work, the problems, and the rational of the work.”

**Response**: We have extended the introduction to provide a more comprehensive background, elucidate the aim of our work, clarify the problems addressed, and outline the rationale behind our research. Additionally, we have incorporated a brief statement of the overall aim of the work and its achievement at the conclusion of the introduction, in line with your suggestions.These revisions have allowed us to provide a more well-rounded introduction, offering readers a clearer understanding of the context, significance, and objectives of our study.

6. “1HNMR and 13C NMR charts should be provided in supplementary data.”

**Response**: We have included the ^1^H NMR and ^13^C NMR spectra of all 19 new derivatives in Supporting Information, presented as a PDF file named S1_File.

7. “Fig 1, 2,3 have poor resolution. "

**Response**: Thank you for bringing this to our attention, and we sincerely apologize for any inconvenience caused. We have now thoroughly checked Figures 1, 2, and 3 using PACE as suggested in the guidelines. Subsequently, we have converted these figures into formats that meet the journal's resolution requirements. You can find the updated and improved figures in the revised manuscript.

8. “The biological target (receptor of enzyme) should be determined. Then docking studies should be carried out against the target receptor.”

Response: We appreciate the reviewer's suggestion regarding the determination of a biological target and conducting docking studies against the target receptor. While we acknowledge the importance of these techniques in understanding the mechanisms of action of compounds, the primary focus of this study was to evaluate the antiproliferative and apoptotic effects of thiosemicarbazone derivatives based on (-)-camphene and R-(+)-limonene in human melanoma cells. The study aimed to provide insights into the potential of these compounds as candidates for further investigation in melanoma therapy. As mentioned in the abstract, we do recognize the need for further investigations, including molecular docking studies, to fully explore the potential of these compounds. However, due to the scope of this study, we were unable to perform these additional analyses at this stage. We will consider the reviewer's suggestion for future research directions.

**Points raised by # Reviewer 2:**

The work exerted is so appreciated. However, some points need to be addressed. So, a major revision may be required to improve the manuscript.

1. “The abstract has to be improved. The abstract should illustrate your work in an attractive way and briefly removing all redundant data.”

**Response**: We sincerely appreciate your kind words and recognition of our efforts in this work. It's encouraging to receive such positive feedback, and we are committed to further enhancing the manuscript to meet the journal's standards. Your valuable input will undoubtedly contribute to the overall quality of our study.

Thank you for your feedback on the abstract. We appreciate your comments, and we want to assure you that we have carefully reevaluated the abstract to make it more concise, reducing its length while retaining its ability to effectively summarize the key findings of our study, as suggested by the previous reviewer. We are committed to further improving the abstract to meet the journal's guidelines and will ensure that it fully illustrates our work in an attractive and concise manner while removing any redundant data as needed.

2. “ Some typos and grammatical errors need to be corrected”

**Response**: Thank you for your feedback regarding the presence of typos and grammatical errors in our manuscript. Following your suggestions, we conducted a thorough review and made the necessary corrections. Additionally, we have further improved the language quality by submitting the manuscript to a professional editing service. We are confident that these steps have significantly enhanced the overall clarity and readability of our work.

3. “In the introduction part, some short paragraphs need to be united into one paragraph.”

**Respons**e: The response to your comment 3 can be found in the subsequent response to comment 4.

4. “The overall introduction is too short. Many points have to be discussed in the introduction parte such as impact of cancer and its gloval statistics, attempts for melanoma treatment, literature of compounds bearin thiosemicarbazone for cancer treatment, and so on...”

**Response**: In response to your suggestion in comment 3, we have consolidated several short paragraphs within the introduction into a single, more cohesive paragraph. This revision has helped improve the flow and readability of the introduction. Regarding your comment 4, we have taken into consideration your valuable points for expanding the introduction. In the revised version, we have provided a more comprehensive introduction that encompasses the global impact of cancer and melanoma, the existing literature on thiosemicarbazones in cancer treatment, and the attempts made in the field of melanoma treatment. We believe this expanded introduction better sets the stage for our study and provides readers with a more thorough understanding of its context.

5. “Please note that in the manuscript, all compounds will be highlighted in bold for readability purposes.” Please remove this sentence!

**Response**: We appreciate your feedback. The sentence requesting the highlighting of all compounds in bold for readability purposes in the manuscript has been removed.

6. “Characterized by one of the coauthors”. Please remove this sentence!

**Response**: Thank you for your comment. The sentence 'Characterized by one of the coauthors' has been removed.

7. “At lines 377-379 and 459-471, please remove highlights.”

**Response**: The highlights at lines 377-379 and 459-471 have been removed, as indicated in the ‘Revised Manuscript with Track Changes’.

8. The resolution of most figures have to be enhanced.”

**Response**: We appreciate your suggestion regarding the enhancement of figure resolution. In response to your comment and a similar one from Reviewer #1, we have taken steps to improve the resolution of all figures. We used the PACE software, as recommended by the journal, which allowed us to enhance the resolution significantly. This improvement ensures that the figures meet the required quality standards.

9. “The experimental chemistry part of the sythesized compounds along with chemical synthesis procedures should be transferred from supplementary to the main manuscript.”

**Response**: Thank you for suggesting the transfer of the experimental chemistry details and synthesis procedures of the synthesized compounds from the Supporting Information to the main manuscript. We have considered your feedback and made the necessary changes. The specific modifications, including the addition of content can be better visualized in the ‘Revised Manuscript with Track Changes’. Consequently, the title of the file named S1_File, which was 'Synthesis and structural characterization of the new thiosemicarbazone derivatives,' has been changed to 'Structural characterization of the new thiosemicarbazone derivatives.'

10. **“**Conclusion part have to be improved.”

**Response**: In our initial submission, we had distinct 'Results', 'Discussion' and ‘Conclusion’ sections. Following your insightful observation, we agree that the content originally in the 'Conclusion' would be more appropriately placed within ‘Discussion’ section. Based on our analysis of articles published in the journal, we understand that the 'Conclusion' section should be concise and to the point. Consequently, in this revised version, we have restructured the manuscript accordingly. We have introduced a new text for the ‘Conclusion’ section, which now aligns with the journal's guidelines for a succinct and clear conclusion. The content from the previous ‘Conclusion’ has been seamlessly integrated into the 'Discussion’ section.

11. “Some references have to be updated.**”**

**Response**: Thank you for highlighting the need for reference updates. In response, we have revised the introduction section, incorporating several references to provide a more comprehensive overview of cancer impact, global statistics, and recent developments in melanoma treatment. Furthermore, in the 'Discussion' section, we have updated the references as follows and included additional ones to enhance the manuscript's quality:

Ferlay J, Colombet M, Soerjomataram I, Parkin DM, Piñeros M, Znaor A, et al. Cancer statistics for the year 2020: an overview. Int J Cancer. 2021;149: 778-789.

Chanda M, Cohen MS. Advances in the discovery and development of melanoma drug therapies. Expert Opin Drug Discov. 2021;16: 1319-1347.

Sung H, Ferlay J, Siegel RL, Laversanne M, Soerjomataram I, Jemal A, et al. Global cancer statistics 2020: GLOBOCAN estimates of incidence and mortality worldwide for 36 cancers in 185 countries. CA Cancer J Clin. 2021;71: 209-249.

Pitucha M, Korga-Plewko A, Czylkowska A, Rogalewicz B, Drozd M, Iwan M, Kubik J, Humeniuk E, Adamczuk G, Karczmarzyk Z, et al. Influence of Complexation of Thiosemicarbazone Derivatives with Cu (II) Ions on Their Antitumor Activity against Melanoma Cells. International Journal of Molecular Sciences. 2021; 22(6):3104.

Shakya B, Yadav PN. Thiosemicarbazones as potent anticancer agents and their modes of action. Mini Rev Med Chem. 2020;20: 638-661.

This update not only addresses the specific references you mentioned but also includes additional pertinent references for a more robust and current literature review.

12. “Where are the charts of IR, 1HNMR, 13CNMR and mas spectrometry?? They have to be shown in the supplementary material.”

**Response**: Regarding your question about the charts of IR, ^1^H NMR, ^13^C NMR, and mass spectrometry, we appreciate your attention to this matter. As mentioned in our cover letter to the editor, we encountered a unique challenge in providing the requested spectra. We were able to include the ^1^H NMR and ^13^C NMR spectra of all 19 new derivatives in the Supporting Information file, named S1_File. The NMR spectra of the 19 new derivatives synthesized are also available in the theses of both authors responsible for their synthesis, and the references to these theses have been included in the 'References' section.

However, for the IR and mass spectra, only 7 IR spectra were present in the thesis of one author, and unfortunately, no mass spectra were available, but the specific characteristic absorption band information for the remaining 8 spectra is presented in tabular format within his thesis. Regrettably, the other author did not include any IR or mass spectra in their thesis, and due to the considerable time that has passed since the defense in 2010, no originals are available. Nevertheless, her thesis does contain all data related to IR absorption bands, as well as molecular íon mass values presented in tabular format.

Hence, we were able to include the ^1^H NMR and ^13^C NMR spectra of all 19 new derivatives, along with the available IR spectra of 7 of them in the Supporting Information file, named S1_File. However, due to the outlined circumstances, we have chosen to exclude mentions of the IR and mass spectra from both the Abstract and the Discussion section.

Consequently, the revised abstract now reads: 'A series of 38 thiosemicarbazone derivatives based on camphene and limonene were evaluated for their antiproliferative activity. Among them, 19 were synthesized and characterized using proton and carbon-13 nuclear magnetic resonance (^1^H and ^13^C NMR).’ In the Discussion section, we have replaced the previous mention with the following text: 'In this study, we synthesized and structurally caracterized 19 thiosemicarbazone compounds based on (-)-camphene using ^1^H and ^13^C-NMR techniques.'

This clarification aims to specifically address your inquiry about the absence of certain spectra in the supplementary material. We acknowledge the importance of these spectra in supporting the scientific content of our manuscript, and we appreciate your diligence in raising this concern.

13. “The work presented is so valued. However, the overall writing patten of the manuscripts has to be improved.”

**Response**: Thank you for your valuable feedback and for acknowledging the value of our work. We have taken your comment regarding the overall writing pattern seriously. In response, we have submitted the manuscript to a professional editing service to improve its overall writing quality.

Finally, we would like to once more extend our gratitude to the editorial team and the reviewers for the thorough examination of our manuscript and their valuable contributions that have enriched our work.

Sincerely,

Lidia
